# Solving non-Hermitian physics for optical manipulation on a quantum computer

**DOI:** 10.1038/s41377-025-01769-2

**Published:** 2025-03-21

**Authors:** Yu-ang Fan, Xiao Li, Shijie Wei, Yishan Li, Xinyue Long, Hongfeng Liu, Xinfang Nie, Jack Ng, Dawei Lu

**Affiliations:** 1 Department of Physics, Southern University of Science and Technology, Shenzhen, 518055 China; 2 Shenzhen Institute for Quantum Science and Engineering, Southern University of Science and Technology, Shenzhen, 518055 China; 3 Department of Physics, The Hong Kong University of Science and Technology, Hong Kong, China; 4 Beijing Academy of Quantum Information Sciences, Beijing, 100193 China; 5 Quantum Science Center of Guangdong-HongKong-Macao Greater Bay Area, Shenzhen, 518045 China; 6 International Quantum Academy, Shenzhen, 518055 China

**Keywords:** Optical manipulation and tweezers, Quantum optics

## Abstract

Intense laser light, with its ability to trap small particles, is providing us unprecedented access to the microscopic world. Nevertheless, owing to its open nature, optical force is nonconservative and can only be described by a non-Hermitian theory. This non-Hermiticity sets such system apart from conventional systems and has offered rich physics, such as the possession of the exceptional points. Consequently, analyzing and demonstrating the dynamics of large optically-bound clusters becomes an intricate challenge. Here, we developed a scalable quantum approach that allows us to predict the trajectories of optically trapped particles and tackle the associated non-Hermitian physics. This approach is based on the linear combination of unitary operations. With this, we experimentally revealed the non-Hermiticity and exceptional point for a single or multiple particles trapped by optical force fields, using a nuclear magnetic resonance quantum processor. Our method’s scalability and stability have offering a promising path for large-scale optical manipulation with non-Hermitian dynamics.

## Introduction

A focus laser beam can grab micro- or nano- particles by minimizing their free energy. This versatile, contactless, and noninvasive technique is known as optical trapping (OT) or optical tweezers^1^, whose applications span a wide spectrum of scientific areas, which include, but are not limited to, physics^2,3^, biology^4–7^, chemistry^8,9^, and nanotechnology^10–14^. Another branch of optical manipulation known as optical binding (OB)^15–17^ studies the light-scattering induced coupling between multiple particles. Be it single or multiple particles, owing to the open nature of the system where light flow in and out while exchanging energy with the particles, it belongs to a class of nonconservative systems^18–23^, which can also be considered as non-Hermitian systems^24,25^. The non-Hermiticity overturns OT and OB, making it completely different from the ordinary Hermitian system. For example, non-Hermitian force fields in OT possess the ability to induce instability in the confined particle, where this instability arises as the system undergoes a transition from stability to instability at the exceptional point (EP)^24,26–28^. Moreover, in OB, the non-Hermiticity prevents light from effectively binding a significant quantity of particles into a stable entity^24^.

In light of the rapid advancements in quantum technologies, there is a growing interest in harnessing the potential of quantum computers for simulating OT and OB systems and addressing the challenges posed by their inherent non-Hermiticity. Quantum computing offers the promise of a substantial speedup, owing to the inherent power of quantum superposition and entanglement. In this work, we leverage the concept of linear combination of unitaries (LCUs)^29–34^ to formulate a quantum approach capable of evolving the system qubits in a manner that faithfully mimics the trajectories of OT and OB particles within a real non-Hermitian environment. As an initial step in our demonstration, we utilize a nuclear magnetic resonance (NMR) quantum processor^35–37^ to tackle the scenario involving a single optically trapped particle. Our findings reveal that quantum evolution closely approximates the theoretical outcomes in the displacement and velocity space. We observe stable OT until the system approaches the EP, from which, it transitions into an unstable structure. Additionally, we conduct numerical simulations involving multiple trapped particles to further validate the effectiveness and scalability of our method.

## Results

### Model

**Fig. 1:**
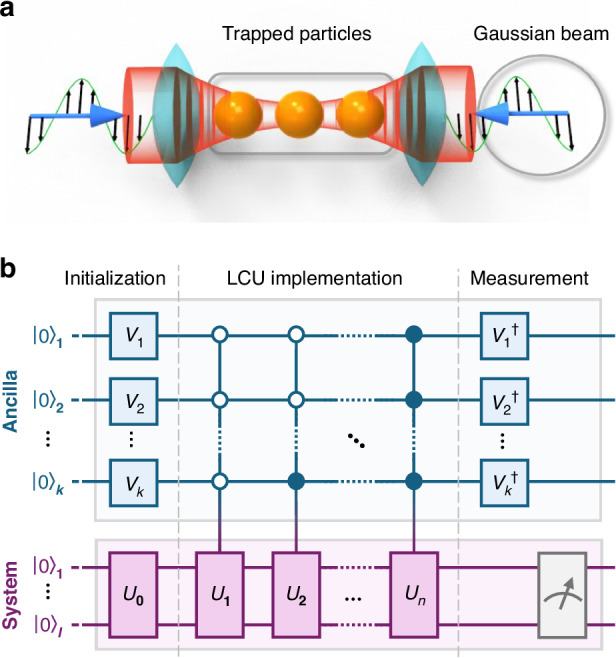
Non-Hermitian optical manipulation and LCU quantum circuit. **a** Schematic depicting optical manipulation of three dielectric particles by a pair of strongly focused counter-propagating Gaussian beam. In this physical scenario, the non-Hermiticity arises from the asymmetric light scattering occurring between different pair of particles (such as the left and middle particles). Consequently, under the influence of light-induced asymmetric force couplings, non-Hermitian physics emerges. **b** General quantum circuit based on LCUs to simulate the OB system. In 3D space, the state of *N* particles can be encoded using $l=\lceil {\log }_{2}(6N)\rceil$ system qubits. To implement the time evolution of the non-Hermitian matrix ${\tilde{{\bf{K}}}}^{{\prime} }$ (refer to the main text), we express it within the LCU framework as ${\tilde{{\bf{K}}}}^{{\prime} }=\mathop{\sum }\nolimits_{i = 1}^{n}{c}_{i}{\tilde{{\bf{U}}}}_{i}$, necessitating $k=\lceil {\log }_{2}n\rceil$ ancillary qubits. The circuit comprises three main segments: Initialization, ${\tilde{{\bf{U}}}}_{0}$ loads the initial state of the particles into the system qubits, and each ${\tilde{{\bf{V}}}}_{i}$ loads the corresponding coefficient *c*_*i*_ into the ancilla qubits. LCU implementation, ${\tilde{{\bf{U}}}}_{i}$ is applied to the system qubits based on the state of the ancilla qubits, achieved through controlled gates. Post-selected measurement, After applying ${\tilde{{\bf{U}}}}_{i}$, ${\tilde{{\bf{V}}}}_{i}^{\dagger }$ restores the ancilla qubits to their initial state. Post-selective measurement of the system qubits occurs when the ancilla qubits are in the state ${\left\vert 0\right\rangle }^{\otimes k}$

In three-dimensional space, let us consider *N* identical spherical particles subjected solely to optical forces, denoted by a vector ${\bf{F}}=({F}_{x}^{1},{F}_{y}^{1},{F}_{z}^{1},\cdots \,,{F}_{x}^{N},{F}_{y}^{N},{F}_{z}^{N})$ with 3*N* components; see Fig. 1a. When the particles are trapped near an equilibrium configuration defined by **F** = **0**, its stability and dynamics are governed by

1 $$m\frac{{\text{d}}^{2}}{{\text{d}}\,{t}^{2}}\Delta {\bf{S}}={\bf{F}}(\Delta {\bf{S}})\approx \tilde{{\bf{K}}}\cdot \Delta {\bf{S}}$$

where *m* denotes the single-particle mass, *t* is the time, Δ**S** = (Δ*x*_1_, Δ*y*_1_, Δ*z*_1_, ⋯ , Δ*x*_*N*_, Δ*y*_*N*_, Δ*z*_*N*_) is the displacement from equilibrium, and *K*_*ij*_ = ∂*F*_*i*_/∂Δ*S*_*j*_ is the matrix element of the 3*N* × 3*N* force matrix $\tilde{{\bf{K}}}$ evaluated at the equilibrium. The general solution to Eq. (1), except right at the EP (which is of measure zero), is given by

2 $$\Delta {\bf{S}}=\mathop{\sum }\limits_{i=1}^{3N}{\alpha }_{i}\Delta {{\bf{S}}}_{0}^{i}{e}^{i{\Omega }_{i}t}$$

where Ω_*i*_ denotes the vibrational frequency corresponding to the *i*-th eigenvalue (*K*_*i*_) of $\tilde{{\bf{K}}}$, *α*_*i*_ is the complex vibration amplitude for the *i*-th mode that is determined by initial conditions, and $\Delta {{\bf{S}}}_{0}^{i}$ is the *i*-th eigenvector of $\tilde{{\bf{K}}}$. The relationship between the eigenvalue *K*_*i*_ and the vibrational frequency Ω_*i*_ is given by ${K}_{i}=-m{\Omega }_{i}^{2}$. In the absence of other interactions, the OT or OB is unstable whenever *K*_*i*_ > 0 or being complex. The first type of instability is trivial; it simply indicates that a particle is located in a locally potential energy maximum. The second type of instability is particularly intriguing due to its emergence from the non-Hermitian nature of this system, which is distinguished by a real and asymmetric (non-Hermitian) $\tilde{{\bf{K}}}$. Here, the occurrence of non-Hermiticity in OT (*N* = 1) can be ascribed to the presence of an optical vortex, which can be induced, for example, through the conversion of spin angular momentum into orbital angular momentum when a circularly polarized Gaussian beam is focused^38^. Moreover, non-Hermiticity can also be induced by the asymmetric light scattering between two arbitrary particles in the case of OB (*N* > 1)^24^, unless it is protected by symmetric properties. Our attention is specifically directed towards comprehending the non-Hermitian characteristics of both OT and OB.

### LCU quantum circuit

As *N* grows large, conducting the eigen-analysis of $\tilde{{\bf{K}}}$ and subsequently obtaining the solution in Eq. (2) becomes a formidable task for classical computers. Alternatively, we suggest employing a quantum computer to simulate the non-Hermitian dynamics of the system, offering a potentially exponential speedup in solving the trajectories of particles. In this section, we outline the fundamental concept of the quantum approach for addressing this challenge, reserving the detailed methodology for the Methods and Supplementary Information.

The initial step is to load the classical information of optically trapped particles into the quantum register. It involves aligning the displacements Δ**S** and velocities $\Delta \dot{{\bf{S}}}$ of the trapped particles into a unified vector ${\bf{Z}}=[\Delta {\bf{S}};\Delta \dot{{\bf{S}}}]$, transforming Eq. (1) into the rewritten form $\dot{{\bf{Z}}}=[\tilde{{\bf{0}}},\tilde{{\mathbb{I}}};\tilde{{\bf{K}}},\tilde{{\bf{0}}}]{\bf{Z}}={\tilde{{\bf{K}}}}^{{\prime} }{\bf{Z}}$, where $\tilde{{\mathbb{I}}}$ represents the 3*N* × 3*N* identity matrix, and ${\tilde{{\bf{K}}}}^{{\prime} }=[\tilde{{\bf{0}}},\tilde{{\mathbb{I}}};\tilde{{\bf{K}}},\tilde{{\bf{0}}}]$ is a 6*N* × 6*N* anti-diagonal block matrix. The solution to this equation is given by ${\bf{Z}}(t)={e}^{{\tilde{{\bf{K}}}}^{{\prime} }t}{\bf{Z}}(0)$. Here, **Z**(0) is determined by the initial condition and can be encoded onto a pure quantum state with up to *l* = ⌈log_2_(6*N*)⌉ qubits.

The crux lies in realizing the time evolution of ${e}^{{\tilde{{\bf{K}}}}^{{\prime} }t}$, a process generally distant from a unitary operator. Traditional quantum gates, ideally unitary, cannot achieve this. To tackle this, we express ${e}^{{\tilde{{\bf{K}}}}^{{\prime} }t}$ using its Taylor expansion form ${e}^{{\tilde{{\bf{K}}}}^{{\prime} }t}={\sum }_{m}{({\tilde{{\bf{K}}}}^{{\prime} }t)}^{m}/m!$. To implement ${({\tilde{{\bf{K}}}}^{{\prime} }t)}^{m}$ individually, we employ a series of quantum circuits with the same LCU structure as shown in Fig. 1b; see Methods. The results obtained by applying these circuits to the vector **Z**(0) are then summed to achieve ${e}^{{\tilde{{\bf{K}}}}^{{\prime} }t}{\bf{Z}}(0)$. In essence, the realization of any *m*-th order term ${({\tilde{{\bf{K}}}}^{{\prime} }t)}^{m}$ relies on the LCU, founded on the fundamental concept that any matrix can be linearly decomposed into a combination of unitaries^30,39^.

Figure 1b illustrates the general quantum circuit for realizing any order of the Taylor expansion of ${e}^{{\tilde{{\bf{K}}}}^{{\prime} }t}$. Taking the first order as an example, ${\tilde{{\bf{K}}}}^{{\prime} }$ can be expressed as ${\tilde{{\bf{K}}}}^{{\prime} }=\mathop{\sum }\nolimits_{i = 1}^{n}{c}_{i}{\tilde{{\bf{U}}}}_{i}$, where *c*_*i*_ are positive coefficients, and ${\tilde{{\bf{U}}}}_{i}$ are unitary operators. The upper *k* qubits represent ancillary qubits staring from ${\left\vert 0\right\rangle }^{\otimes k}$, and ${\tilde{{\bf{V}}}}_{i}\,(1\le i\le k)$ are unitary operations preparing the ancillary qubits to the pure state ${[\sqrt{{c}_{1}},\sqrt{{c}_{2}},\ldots ,\sqrt{{c}_{n}}]}^{{\rm{T}}}/{\mathbb{N}}$, with ${\mathbb{N}}=\sqrt{{\sum }_{i}{c}_{i}}$ as the normalization condition. Thus, at most $k=\lceil {\log }_{2}n\rceil$ ancillary qubits suffice to store these coefficients *c*_*i*_. The lower *l* system qubits record the classical information of **Z**(0) and undergo subsequent evolution controlled by the ancillary qubits. The first gate ${\tilde{{\bf{U}}}}_{0}$ prepares the system qubits into the normalized quantum state **Z**(0)/∣∣**Z**(0)∣∣, where ∣∣ ⋅ ∣∣ denotes the modulus operation.

The subsequent *n* gates, denoted by ${\tilde{{\bf{U}}}}_{i}\,(1\le i\le n)$, operate under the control of the state of the *k* ancillary qubits. Examining the input of the *k* ancillary qubits reveals that these controlled operations entangle the ancillary and system qubits, with the coefficient *c*_*i*_ incorporated into the entire entangled state; see Methods. The post-processing operator ${\tilde{{\bf{V}}}}_{j}^{\dagger }$ is applied to restore the *k* ancillary qubits to their initial state, after which we can perform post-selective measurement of the system qubits when the ancilla qubits are ${\left\vert 0\right\rangle }^{\otimes k}$. The system’s final state becomes: $\left\vert \Psi \right\rangle =\frac{1}{{\mathbb{M}}}\sum {c}_{i}{\tilde{{\bf{U}}}}_{i}{\bf{Z}}(0)=\frac{1}{{\mathbb{M}}}{\tilde{{\bf{K}}}}^{{\prime} }{\bf{Z}}(0)$, where ${\mathbb{M}}=\sum {c}_{i}| | {\bf{Z}}(0)| |$ serves as the normalization factor. Therefore, we can acquire the results of the first-order Taylor expansion ${\tilde{{\bf{K}}}}^{{\prime} }{\bf{Z}}(0)$, subject to a certain normalized condition. The circuit for obtaining other orders of the Taylor expansion follows a similar structure, and the technical details are elaborated in Supplementary Information.

### Quantum simulation of a single optically trapped particle

To illustrate the quantum approach outlined above, we conduct experiments utilizing nuclear spins through the NMR technique^33,40^. The quantum processor employed comprises crotonic acid dissolved in *d*_6_-acetone, where ^13^C and ^1^H nuclei serve as qubits. These experiments are carried out at room temperature using a Bruker 600 MHz NMR spectrometer. The internal Hamiltonian governing the system is expressed as ${{\mathcal{H}}}_{{\rm{NMR}}}=-\pi {\sum }_{i}{\nu }_{i}{\sigma }_{z}^{i}+\pi {\sum }_{i < j}{J}_{ij}{\sigma }_{z}^{i}{\sigma }_{z}^{j}/2,$ where ${\sigma }_{z}^{i}$ signifies the Pauli matrix of the *i*-th spin, *ν*_*i*_ is the chemical shift relative to the reference frequency, and *J*_*ij*_ denotes the coupling strength between the *i*-th and *j*-th spins^41^. Specific values for *ν*_*i*_ and *J*_*ij*_ can be found in Methods. Moreover, transverse radio-frequency pulses can be applied to enact single-qubit rotations^42^.

The model employed in the first simulation involves a single optically trapped particle, exhibiting non-Hermitian dynamics. Owing to the presence of symmetry properties, the force matrix $\tilde{{\bf{K}}}$ in OT for a single particle can be block-diagonalized into two distinct components: a 2 × 2 real matrix ${\tilde{{\bf{K}}}}_{{\rm{OT}}}$ associated with transverse 2D motion, and a real scalar representing decoupled longitudinal harmonic motion. For simplicity, our attention is directed solely towards the 2 × 2 matrix ${\tilde{{\bf{K}}}}_{{\rm{OT}}}$. Upon suitable rotation, without loss of generality, this matrix assumes the form

3 $${\tilde{{\bf{K}}}}_{{\rm{OT}}}=\left[\begin{array}{cc}a+b&g\\ -g&a-b\end{array}\right]$$

where *a* signifies the average trap stiffness, *b* represents half the level spacing between the two trap stiffnesses, and *g* originates from the nonconservative force. This equation characterizes an anisotropic harmonic oscillator subject to a nonconservative force and is applicable in both 2D and 3D cases^24^.

As delineated by Eq. (2), the stability of this model is dictated by the eigenvalues of ${\tilde{{\bf{K}}}}_{{\rm{OT}}}$:

4 $${K}_{\pm }=-m{\Omega }^{2}=\left\{\begin{array}{ll}a\pm \left\vert \sqrt{{b}^{2}-{g}^{2}}\right\vert ,\quad &| g|\, < \,| b| \\ a,\quad &| g| =| b| \\ a\pm i\left\vert \sqrt{{b}^{2}-{g}^{2}}\right\vert ,\quad &| g| \,> \,| b| \end{array}\right.$$

We exclusively consider Re(*K*_±_) < 0, as the system is otherwise unstable, regardless of the value of *g*. At *g* = 0, the system exhibits simple harmonic oscillation about the zero-force position. For nonzero *g*, the system is non-Hermitian. As evident from Eq. (3), a non-zero *b* introduces an anisotropy between the two axes, creating a level spacing in the vibration levels. This establishes a rotational barrier for the particle, as it tends to align with one of the axes. When ∣*g*∣ < ∣*b*∣, the nonconservative force associated with *g* fails to overcome the barrier *b*. The modes experience twisting by *g*, inducing non-orthogonality in the eigenmodes; otherwise, they closely resemble harmonic oscillators. For ∣*g*∣ > ∣*b*∣, a conjugate pair of vibration frequencies, Ω_+_ and Ω_−_, emerge, corresponding to stable spiral-in and unstable spiral-out motions, respectively. The nonconservative force can now drive the particle to revolve, and gaining energy (and thus vibrtaion amlpitude) on every loop it moves. The point, where ∣*g*∣ = ∣*b*∣, is the EP, which is a singular defective point for the non-Hermitian matrix.

**Fig. 2:**
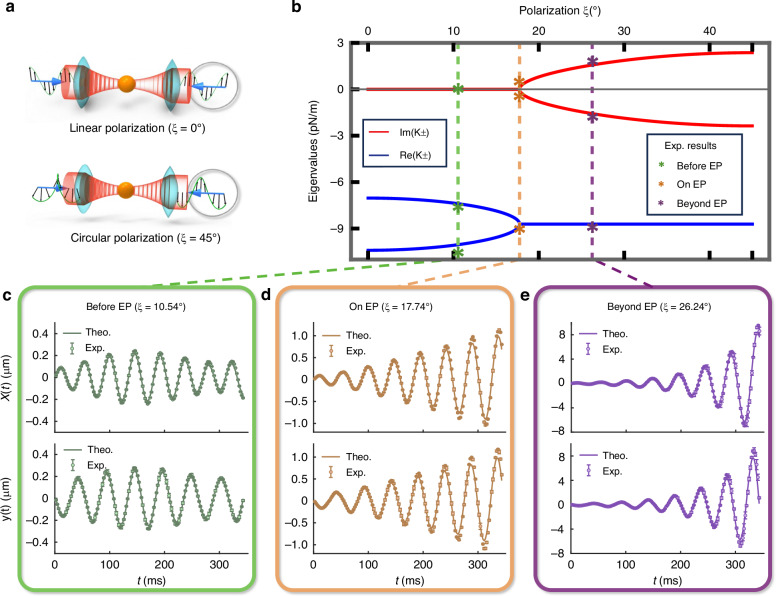
Experimental results of simulating a single optically trapped particle. **a** Considering a dielectric particle captured by a pair of strongly focused, counter-propagating Gaussian beams, the non-Hermiticity increases when changing the polarization ($\widehat{{\bf{p}}}=\widehat{{\bf{x}}}\,\text{cos}\,\xi +i\widehat{{\bf{y}}}\,\text{sin}\,\xi$) of the beams from linear (*ξ* = 0^∘^) to circular (*ξ* = 45^∘^). **b** Plot of eigenvalues as a function of polarization. The EP occurs at *ξ* = 17.74^∘^, beyond which the eigenvalues become complex, implying instability. We highlight three typical points: particle trapped (*ξ* = 10.54^∘^, green), on the EP (*ξ* = 17.74^∘^, orange), and beyond the EP (*ξ* = 26.24^∘^, purple). Colored stars indicate experimentally determined eigenvalues, which align well with theoretical values. **c**–**e** Experimental results of displacement dynamics versus time. For the three cases, stable, diverging, and unstable oscillations are observed, respectively, consistent with predictions from non-Hermitian dynamics theory. The curves are numerical simulations and circles are experimental results. The error bars are derived from repetition of multiple experimental results by measuring the final states (see Supplementary Information)

The above model can be applied to a dielectric particle captured by a pair of strongly focused, counter-propagating Gaussian beams with an overlapping focus. A solitary particle, featuring a refractive index of 1.45 and a radius of 0.5 *μ*m, is trapped at the waist of the counter-propagating Gaussian beam in a vacuum. Each beam possesses a power of 1 mW, a wavelength *λ* of 1064 nm, and a numerical aperture of 0.9. In this specific scenario, the polarization ($\widehat{{\bf{p}}}=\widehat{{\bf{x}}}\,\text{cos}\,\xi +i\widehat{{\bf{y}}}\,\text{sin}\,\xi$) of the Gaussian beam transits from linear (*ξ* = 0^∘^) to circular (*ξ* = 45^∘^), as shown in Fig. 2a. During this process, the non-Hermitian characteristic (*g* in the ${\tilde{{\bf{K}}}}_{{\rm{OT}}}$) arising from the conversion of angular momentum from spin to orbital ones^38^, while the beams are being focused, becomes more pronounced and exhibits an increasing trend with respect to *ξ*. Moreover, an EP is observed when ∣*g*∣ = ∣*b*∣, as marked with dashed orange line in Fig. 2b. In the complete spectrum of eigenvalues (*K*_±_ in Fig. 2b) as a function of polarization (characterized by *ξ*), three key points are highlighted, where the particle is trapped stably (*ξ* = 10.54^∘^, green, real *K*_±_), on the EP (*ξ* = 17.74^∘^, orange), and beyond the EP (*ξ* = 26.24^∘^, purple, complex *K*_±_). To study the time-varying particle trajectories of the optically trapped particle, we have selected *x*(0) = − 15 nm and *y*(0) = 25 nm, serving as the starting point, for the initial coordinates. And we load this normalized input vector that encodes the initial information of displacements and velocities into the quantum register using an optimized shaped pulses (see Supplementary Information).

The subsequent phase involves the implementation of quantum gates, depicted in Fig. 1b. Employing gradient-based optimization, we achieve single-qubit rotations and controlled gates, each exceeding 0.995 fidelity in numerical simulations. Quantum state tomography is performed on the system qubits, followed by maximum likelihood approximation to extract a pure quantum state that is the closest to the measured density matrix. The corresponding displacements and velocities at time *t* are then derived from the final measured state. Modulating the evolution time *t* allows us to capture the oscillation dynamics of this single-particle OT model.

Figure 2c–e display the experimental results (dots) of displacements, *x*(*t*) and *y*(*t*), over time alongside numerical simulations (lines) for the three cases: trapping (*ξ* = 10.54^∘^), on the EP (*ξ* = 17.74^∘^), and beyond the EP (*ξ* = 26.24^∘^). In the trapped case (green), displacements in the *x*-*y* plane exhibit stable oscillations, with fitted vibration frequencies ${\Omega }_{1}^{+}=0.104$ and ${\Omega }_{1}^{-}=0.075$ closely aligned with theoretical predictions which are 0.101 and 0.074. When on the EP (orange), a diverging oscillation is observed. The fitted vibration frequencies ${\Omega }_{2}^{\pm }=0.898\pm 0.037i$ deviate slightly due to experimental imperfections, compared to theoretical predictions (both at 0.878). After surpassing the EP (purple), unstable diverging oscillation occurs. Consequently, large imaginary parts emerge in the fitted vibration frequencies ${\Omega }_{3}^{\pm }=0.888\pm 0.175i$, in good agreement with theoretical values 0.872 ± 0.157*i*. This case corresponds to the non-Hermitian dynamics of the single-particle OT model. The experimentally determined eigenvalues are illustrated in Fig. 2b using colored stars for enhanced visibility. Further error analysis can be found in the Supplementary Information.

**Fig. 3:**
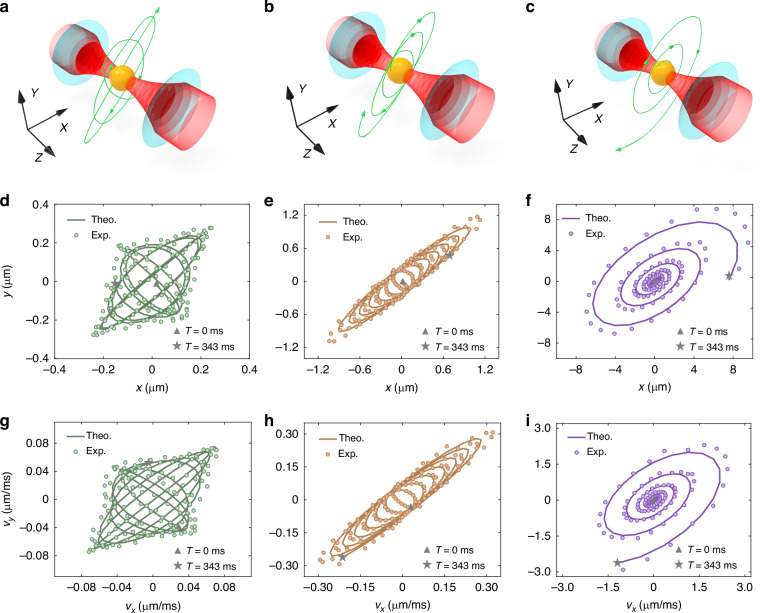
Dynamics for optically trapped particle, on the EP, and beyond the EP. From left to right, the figures correspond to the cases of optical trapping (*ξ* = 10.54^∘^, green), on the EP (*ξ* = 17.74^∘^, orange), and beyond the EP (*ξ* = 26.24^∘^, purple), respectively. **a**–**c** Schematic trajectories of the particle on the *x*-*y* plane manipulated by the strongly focused Gaussian beam. **d**–**f** Experimental results and numerical simulations of the trajectories, and (**g**–**i**) velocities of the particle on the *x*-*y* plane

Additionally, we measure displacements and velocities along the *x* and *y* directions and plot the trajectories of the single particle in the 2D plane. Figure 3d–f showcase the remarkable proximity between experimental results and theoretical predictions. The three cases distinctly depict stable oscillation, diverging oscillation, and rapid oscillation when the vibration frequencies are within, on, and beyond the EP, thereby revealing the non-Hermitian property within this model. Figure 3g–i depict the velocity for the three cases. When comparing with the displacement oscillations, the velocity oscillations exhibit slightly different shapes and phases, with an approximate *π*/2 phase shift, showcasing the characteristic behavior of harmonic oscillations.

### Numerical simulation of an optically bound three-particle system

**Fig. 4:**
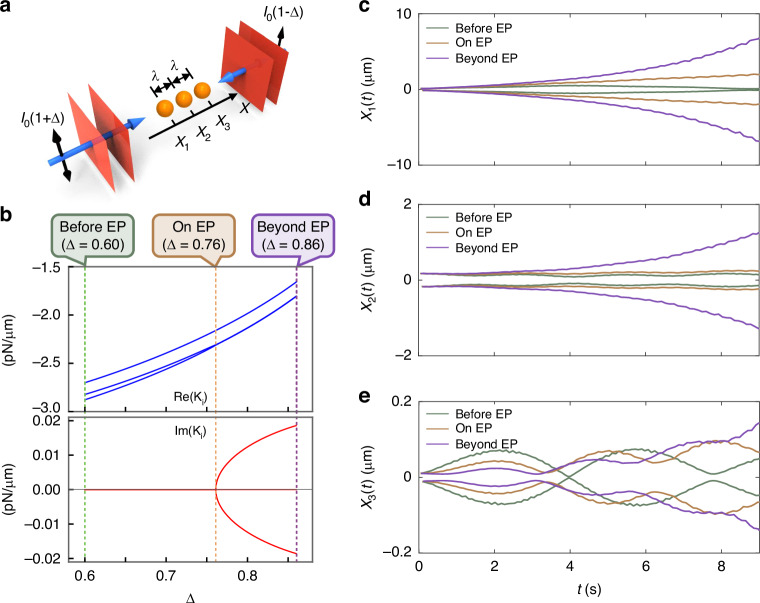
Numerical simulations of the three-particle optical trapping model. **a** Schematic of the model. **b** Real and imaginary parts of the eigenvalues of the force matrix when Δ, which characterizes the intensity difference between the two plane waves, ranges from 0.60 to 0.86. The EP emerges at Δ = 0.76. **c**–**e** Displacements of the three particles over time before, on and beyond the EP. All three particles exhibit stable binding before the EP (Δ = 0.60), linear divergence at the EP (Δ = 0.76), and instability beyond the EP (Δ = 0.86)

The LCU quantum circuit can be extended to address the non-Hermitian physics of a many-particle OB scenario. Here, we are examining a configuration of three dielectric particles that are optically bound and arranged in 1D space, as illustrated in Fig. 4a. The two counter-propagating plane waves bind the three identical particles (with a refractive index of 1.15 and a radius of 0.25 *μ*m) in a chain with a separation between neighboring particles equivalent to one wavelength approximately. The intensities of the two plane waves are deliberately chosen as *I*_1_ = *I*_0_(1 + Δ) and *I*_2_ = *I*_0_(1 − Δ), where the symmetry is broken when Δ ≠ 0. The broken symmetry leads to an enhanced non-Hermiticity. Upon increasing the Δ (non-Hermiticity), we observe the transition of the eigenvalues of $\tilde{{\bf{K}}}$ (from real to complex eigenvalues) and the existence of EP (Δ = 0.76), as shown in Fig. 4b, c.

In the model mentioned above, the movement of the three particles in the *x*-direction is independent of their movement in the transverse directions. As a result, we consider a total of six degrees of freedom, including three displacements and three velocities. Consequently, the dynamics of the entire system can be encapsulated within a 6 × 6 matrix ${\tilde{{\bf{K}}}}^{{\prime} }$, necessitating a three-qubit quantum register to encode the system’s state. Additionally, to efficiently decompose the non-unitary operation using the LCU approach, an additional four ancillary qubits are required. We utilize a 7-qubit crotonic acid molecule comprising three ^1^H and four ^13^C spins for the experimental setup. However, due to the implementation of many Toffoli-like gates as depicted in Fig. 1b, the entire sequence becomes time-intensive, exceeding the relaxation times of the qubits (Methods). Therefore, we perform numerical experiments based on the molecular structure and parameters (Supplementary Information). Below, we present the results obtained from these numerical simulations.

Figure 4c–e display the displacements of the three particles over time. In this representation, we have omitted the rapid oscillations and instead plotted the envelope to highlight the distinct dynamics of the particles for the three cases. Initially, the three particles are separated by one wavelength and continue to exhibit modulated behavior over time. Trajectories of the three particles over time are depicted. The distinct behaviors observed include stable oscillation, diverging oscillation, and unstable oscillation, corresponding to scenarios where the particle radius is within, on, and beyond the EP. This observation underscores the non-Hermitian nature inherent in this three-particle OT model, highlighting the effectiveness of our method in handling particles under more complex conditions.

## Discussion

Optical forces, inherently nonconservative due to their open nature, defy description by Hermitian theories. As the scale of optically-bound clusters increases, analyzing and demonstrating their dynamics becomes increasingly challenging. We propose leveraging quantum computing to accurately simulate the trajectories of optically trapped or bound particles within a real non-Hermitian environment. This approach transforms the non-Hermitian physics problem into an eigendecomposition task using LCUs. To validate the experimental efficacy, we use nuclear spins to replicate the trajectories of a single optically-trapped particle. Our demonstration reveals a stable-to-unstable transition near the EP. Given the limited lifetime of qubits, we further verify the many-particle OB scenario via numerical simulations, demonstrating analogous non-Hermitian dynamics and confirming the approach’s scalability.

Further optimizations are required for our quantum approach. While the method can be faithfully executed using a universal quantum computer, treating high-dimensional tasks poses challenges with current techniques. The LCU method demands numerous ancillary qubits and complex control operations on system qubits, which are difficult to realize with high fidelity using state-of-the-art methods^43^. Simplifying the LCU decomposition is crucial to make it feasible on existing quantum devices^44^. Additionally, our current focus on solving particle trajectories using a quantum register may not represent the most efficient approach to solving the non-Hermitian physics problem in OT and OB. We anticipate a direct solution to eigenvalues and eigenvectors inspired by this method.

In conclusion, our work pioneers a new attempt to leverage quantum computers for solving non-Hermitian physics problems in optical manipulation and other similar classical scenarios. The scalability and stability have been confirmed through single-particle experiments and many-particle numerical simulations, offering a promising path for large-scale optical manipulation with non-Hermitian dynamics.

## Materials and methods

### Computing the electromagnetic field for single or multiple spherical particles

The Mie scattering theory provides a semi-analytical solution to the light scattering problem by a single spherical particle^45^. (1) The wave equations are solved separately inside and outside the sphere using spherical coordinates, yielding a series of vector spherical wave functions. (2) The incident, internal, and scattered fields are expanded in terms of these vector spherical wave functions. (3) Since the incident field is known, its expansion coefficients can be directly obtained. (4) The internal and scattered coefficients are then determined by applying the boundary conditions at the surface of the sphere. Such an approach is “exact” in the sense that no approximation is made, except the finite truncation of the expansion series. For multiple spherical particles, to apply the boundary conditions over the surface of all particles, their scattered fields must be connected using the vector translation-addition theorem (see Supplementary Information for details).

### Computing the optical force

Once the total electromagnetic field is solved by using the Mie thoery described above, one can then apply the time averaged Maxwell stress tensor (${\langle \overleftrightarrow{{\bf{T}}}\rangle }_{{\rm{t}}}$) to calculate the time averaged optical force^46^: ${\bf{F}}={\oint }_{{\rm{S}}}{\langle \overleftrightarrow{{\bf{T}}}\rangle }_{{\rm{t}}}\cdot {\rm{d}}{\bf{a}}$.

### To find equilibrium positions and compute force matrix

To find the equilibrium positions, we employ dynamic simulations that advance the particle motion over time within a fictitious medium featuring damping. These simulations utilize an efficient integrator. The simulation concludes when we detect an equilibrium state where the optical forces acting on all particles reach zero. Near equilibrium, we calculated the force matrix $\tilde{{\bf{K}}}$ with 5-point finite difference method, employing the definition *K*_*ij*_ = ∂*F*_*i*_/∂Δ*S*_*j*_.

### Sample

**Fig. 5:**
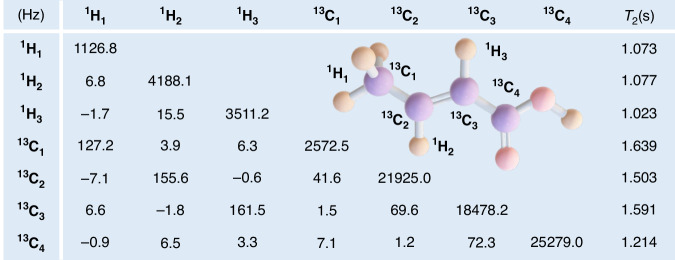
Molecular structure and parameters of the NMR quantum processer. The relative frequencies (diagonal) as well as *J*-coupling values (off-diagonal) between qubits and their dephasing time *T*_2_ are listed in the table

The NMR sample used in our experiment is ^13^C-labeled crotonic acid dissolved in acetone-*d*_6_. We selected three (^13^C_1_, ^13^C_2_, and ^13^C_3_ in Fig. 5) out of the four ^13^C spins for the single-particle experiment. Each qubit can be controlled using radio-frequency (r.f.) pulses. All experiments were conducted on a Bruker AVANCE 600 MHz spectrometer at room temperature.

The molecular structure of crotonic acid is illustrated in Fig. 5. The ^13^C and ^1^H spins in the sample have distinct Larmor frequencies under the strong static magnetic field *B*_0_ = 14.1 T due to their different gyromagnetic ratios. The *J*-coupling interactions between the spins enable two-qubit entangling gates. We provide the relative frequencies and *J*-coupling values between qubits in Fig. 5. In the rotating frame with a reference frequency *ν*_0_, the internal Hamiltonian is represented as

$${{\mathcal{H}}}_{{\rm{NMR}}}=-\pi \sum _{i}{\nu }_{i}{\sigma }_{z}^{i}+\pi \sum _{i < j}{J}_{ij}{\sigma }_{z}^{i}{\sigma }_{z}^{j}/2$$

where ${\sigma }_{z}^{i}$ denotes the Pauli matrix of the *i*-th spin, *ν*_*i*_ is the chemical shift relative to the reference frequency, and *J*_*ij*_ is the coupling strength between the *i*-th and *j*-th spins. The central frequency for the controlled r.f. pulses applied to the ^13^C spins matches the reference frequency *ν*_0_.

To design single-qubit gates, we utilize r.f. shaped pulses that are resonant with the reference frequency *ν*_0_. The control Hamiltonian is described as

$${{\mathcal{H}}}_{{\rm{ctrl}}}={\omega }_{1}(\cos \phi \sum _{i}{\sigma }_{x}^{i}+\sin \phi \sum _{i}{\sigma }_{y}^{i})/2$$

Here, *ω*_1_ is the amplitude of the control field, and *ϕ* is the phase. They can be adjusted over time to create a shaped pulse. The time evolution dynamics, combining the internal and control Hamiltonians within one segment of the shaped pulse, is given by $\exp [-i({{\mathcal{H}}}_{{\rm{NMR}}}+{{\mathcal{H}}}_{{\rm{ctrl}}})\tau ]$, where *τ* represents the time step. By manipulating *ω*_1_, *ϕ*, and *τ* during the pulse design, we can achieve arbitrary single-qubit rotations. This pulse optimization is accomplished using a gradient-based optimal control technique, with simulated fidelities exceeding 0.995. Two-qubit gates are implemented through the free evolution of *J*-couplings, during which unwanted qubits are decoupled from the target qubits (Supplementary Information), and further optimized using gradient-based methods.

The system initialization is achieved using the pseudo-pure state (PPS) technique, which prepares a thermal equilibrium state to the PPS. Measurement is performed via full quantum state tomography using a set of readout pulses. Further details regarding these procedures are provided in the Supplementary Information.

### LCU decomposition for the single-particle experiment

In the single-particle case, the force matrix is block-diagonalized into a 2 × 2 real matrix ${\tilde{{\bf{K}}}}_{{\rm{OT}}}$ as shown in Eq. (3) and a real scalar corresponding to simple harmonic motion. We rewrite it into the generalized force matrix ${\tilde{\bf{K}}}^{\prime} = [\tilde{\mathbf{0}},\tilde{\mathbb{I}};\tilde{\mathbf{K}},\tilde{\mathbf{0}}]$. To realize the time evolution of ${e}^{{\tilde{{\bf{K}}}}^{{\prime} }t}$, we express it using its Taylor expansion form ${e}^{{\tilde{{\bf{K}}}}^{{\prime} }t}={\sum }_{m}{({\tilde{{\bf{K}}}}^{{\prime} }t)}^{m}/m!$. Here, we take the first two ranks of this Taylor expansion. Both ${\tilde{{\bf{K}}}}^{{\prime} }$ and ${\tilde{{\bf{K}}}}^{{\prime} 2}$ can be divided into two respective unitary matrices. Taking ${\tilde{{\bf{K}}}}^{{\prime} 2}$ as an example (the case of ${\tilde{{\bf{K}}}}^{{\prime} }$ can be found in the Supplementary Information), its form is

$$\begin{array}{lll}{\tilde{{\bf{K}}}}^{{\prime} 2}&=&{\tilde{{\bf{I}}}}_{2}\otimes \left[\begin{array}{cc}a+b&g\\ -g&a-b\end{array}\right]\\&=&{\tilde{{\bf{I}}}}_{2}\otimes \left[\begin{array}{cc}a&g\\ -g&a\end{array}\right]+b{\tilde{{\bf{I}}}}_{2}\otimes \left[\begin{array}{cc}1&0\\ 0&-1\end{array}\right]\end{array}$$

where *a* signifies the average trap stiffness, *b* represents half the level spacing between the two trap stiffnesses, *g* originates from the nonconservative force, and ${\tilde{{\bf{I}}}}_{2}$ represents the 2 × 2 identity matrix.

It can be represented by a linear combination of two unitaries, i.e., ${\tilde{{\bf{K}}}}^{{\prime} 2}={c}_{1}{\tilde{{\bf{U}}}}_{1}+{c}_{2}{\tilde{{\bf{U}}}}_{2}$, where

$${\tilde{{\bf{U}}}}_{1}=\frac{1}{\sqrt{{a}^{2}+{g}^{2}}}{\tilde{{\bf{I}}}}_{2}\otimes \left[\begin{array}{cc}a&g\\ -g&a\end{array}\right],{\tilde{{\bf{U}}}}_{2}={\tilde{{\bf{I}}}}_{2}\otimes \left[\begin{array}{cc}1&0\\ 0&-1\end{array}\right]$$

The coefficients satisfy ${c}_{1}=\sqrt{{a}^{2}+{g}^{2}}$ and *c*_2_ = *b*.

As shown in Fig. 1b, to implement the LCU circuit, we require one ancillary qubit (*k* = 1) and two system qubits (*l* = 2). Initially, $\tilde{{\bf{V}}}$ prepares the ancillary qubit to the state $[\sqrt{{c}_{1}};\sqrt{{c}_{2}}]/\sqrt{{c}_{1}+{c}_{2}}$, and ${\tilde{{\bf{U}}}}_{0}$ prepares the two system qubits into the normalized input state **Z**(0)/∣∣**Z**(0)∣∣. Therefore, the initial state of the entire system is given by

5 $$\left\vert {\Psi }_{1}\right\rangle =\frac{1}{\sqrt{{c}_{1}+{c}_{2}}}\left[\begin{array}{c}\sqrt{{c}_{1}}\\ \sqrt{{c}_{2}}\end{array}\right]\otimes \frac{{\bf{Z}}(0)}{| | {\bf{Z}}(0)| | }$$

The following two controlled operations create entanglement between the ancillary and system qubits. In this circuit, the first controlled operation implies that the unitary operation ${\tilde{{\bf{U}}}}_{1}$ is applied to the two system qubits only when the ancillary qubit is $\left\vert 0\right\rangle$. Similarly, the second controlled operation indicates that ${\tilde{{\bf{U}}}}_{2}$ is applied to the system qubits when the ancillary qubit is $\left\vert 1\right\rangle$. Here, we can clearly see the role played by the ancillary qubit—to selectively apply ${\tilde{{\bf{U}}}}_{1}$ and ${\tilde{{\bf{U}}}}_{2}$ based on the state of the ancillary qubit, contributing to the state represented by ${c}_{1}{\tilde{{\bf{U}}}}_{1}+{c}_{2}{\tilde{{\bf{U}}}}_{2}$ scaled by a manageable constant. After applying these two controlled operations, the quantum state of the system is

6 $$\left\vert {\Psi }_{2}\right\rangle =\frac{\sqrt{{c}_{1}}\left\vert 0\right\rangle }{\sqrt{{c}_{1}+{c}_{2}}}\otimes \frac{{\tilde{{\bf{U}}}}_{1}{\bf{Z}}(0)}{| | {\bf{Z}}(0)| | }+\frac{\sqrt{{c}_{2}}\left\vert 1\right\rangle }{\sqrt{{c}_{1}+{c}_{2}}}\otimes \frac{{\tilde{{\bf{U}}}}_{2}{\bf{Z}}(0)}{| | {\bf{Z}}(0)| | }$$

The final operator ${\tilde{{\bf{V}}}}^{\dagger }$ is the conjugate transpose of $\tilde{{\bf{V}}}$, which rotates the ancillary qubit back to $\left\vert 0\right\rangle$. Hence, the final state of the entire system is

7 $$\left\vert {\Psi }_{3}\right\rangle =\left\vert 0\right\rangle \otimes \frac{{c}_{1}{\tilde{{\bf{U}}}}_{1}+{c}_{2}{\tilde{{\bf{U}}}}_{2}}{{c}_{1}+{c}_{2}}\frac{{\bf{Z}}(0)}{| | {\bf{Z}}(0)| | }$$

Therefore, by post-selecting the ancillary qubit when it is $\left\vert 0\right\rangle$, the system qubits undergo the dynamics $({c}_{1}{\tilde{{\bf{U}}}}_{1}+{c}_{2}{\tilde{{\bf{U}}}}_{2}){\bf{Z}}(0)$, which corresponds to the second-order Taylor expansion term ${\tilde{{\bf{K}}}}^{{\prime} 2}\,\text{Z}\,(0)$.

### Settings for the three-particle simulation

For the three-particle case, where three dielectric particles are arranged in 1D space, the force matrices ${\tilde{{\bf{K}}}}_{i}$ when the system is before the EP (*i* = 1), on the EP (*i* = 2), and beyond the EP (*i* = 3) are represented by

$${\tilde{{\bf{K}}}}_{1}=\left[\begin{array}{ccc}-2.83\times 1{0}^{-6}&+8.16\times 1{0}^{-8}&+3.12\times 1{0}^{-8}\\ +1.32\times 1{0}^{-8}&-2.87\times 1{0}^{-6}&+8.99\times 1{0}^{-8}\\ +1.16\times 1{0}^{-8}&+1.02\times 1{0}^{-8}&-2.73\times 1{0}^{-6}\end{array}\right]\frac{{\rm{N}}}{{\rm{m}}}$$

$${\tilde{{\bf{K}}}}_{2}=\left[\begin{array}{ccc}-2.30\times 1{0}^{-6}&+9.34\times 1{0}^{-8}&+5.66\times 1{0}^{-8}\\ +3.92\times 1{0}^{-9}&-2.31\times 1{0}^{-6}&+1.02\times 1{0}^{-7}\\ +6.25\times 1{0}^{-9}&+1.92\times 1{0}^{-9}&-2.17\times 1{0}^{-6}\end{array}\right]\frac{{\rm{N}}}{{\rm{m}}}$$

$${\tilde{{\bf{K}}}}_{3}=\left[\begin{array}{ccc}-1.80\times 1{0}^{-6}&+1.01\times 1{0}^{-7}&+6.00\times 1{0}^{-8}\\ -1.19\times 1{0}^{-9}&-1.80\times 1{0}^{-6}&+1.10\times 1{0}^{-7}\\ +3.04\times 1{0}^{-9}&-2.53\times 1{0}^{-9}&-1.66\times 1{0}^{-6}\end{array}\right]\frac{{\rm{N}}}{{\rm{m}}}$$

Initially, we expand the above matrices to the generalized force matrices ${\tilde{{\bf{K}}}}_{i}^{{\prime} }$, which are 6 × 6. Therefore, we require three system qubits (*l* = 3) to encode the dynamics information (see Supplementary Information). Furthermore, the LCU decomposition of these matrices requires *n* = 13 unitaries (see Supplementary Information), hence we need four ancillary qubits (*k* = 4) to implement the LCU circuit in Fig. 1b. In total, the three-particle case corresponds to a 7-qubit experiment.

We utilize the crotonic acid molecule (Fig. 5), comprising three ^1^H and four ^13^C spins, to form the 7-qubit quantum register. On average, the pulse length of single-qubit rotations $\tilde{{\bf{V}}}$ is about 2 ms, and the pulse length of the controlled unitaries $\tilde{{\bf{U}}}$ is beyond 100 ms, according to the interaction strengths (~100 Hz) between the nuclear spins. The total LCU circuit length is estimated to be over 1.4 s, which is much longer than the *T*_2_ relaxation times (~1 s) of the qubits in the sample; see Fig. 5. Due to this constraint, we perform numerical simulations based on the molecular parameters for the three-particle case, instead of carrying out the NMR experiments.

The simulation starts when the three particles are slightly away from their equilibrium, i.e., [−0.12, −0.18, −0.01] *μ*m. Similar to the single-particle case, the second-order Taylor expansion is considered. We have simulated the entire LCU quantum circuit in the absence of decoherence, and non-Hermitian dynamics beyond the EP is observed, as described in the main text.

## Supplementary information

Supplementary Information

## Data Availability

The data supporting the plots within this article and other findings of this study are available from the corresponding authors on a reasonable request.
