# Supplementary material for: Solving non-Hermitian physics for optical manipulation on a quantum computer
Source: Light Sci Appl. 2025 Mar 21;14:132. doi: 10.1038/s41377-025-01769-2 (PMC11928612; doi:10.1038/s41377-025-01769-2)
Supplement: Supplementary file 1 — Supplementary Information [file 41377_2025_1769_MOESM1_ESM.pdf]

Supplementary Information for "Solving Non-Hermitian Physics for Optical Manipulation on a Quantum Computer"

Yu-ang Fan,[1,2,*] Xiao Li,[1,3,*] Shijie Wei,[4,*] Yishan Li,[1,2] Xinyue Long,[1,5] Hongfeng Liu,[1] Xinfang Nie,[1,2,5] Jack Ng,[1,†] and Dawei Lu[1,2,5,6,‡]

[1]*Department of Physics, Southern University of Science and Technology, Shenzhen 518055, China*
[2]*Shenzhen Institute for Quantum Science and Engineering, Southern University of Science and Technology, Shenzhen 518055, China*
[3]*Department of Physics, The Hong Kong University of Science and Technology, Hong Kong, China*
[4]*Beijing Academy of Quantum Information Sciences, Beijing 100193, China*
[5]*Quantum Science Center of Guangdong–HongKong–Macao Greater Bay Area, Shenzhen 518045, China*
[6]*International Quantum Academy, Shenzhen 518055, China*

GENERALIZED MIE THEORY FOR CLUSTERS OF SPHERICAL PARTICLES

Here, we provide a concise overview of the standard generalized Mie theory for a cluster of spherical particles. This well-established theory has been extensively discussed in the literature, and we have adopted a notations that closely resemble those in reference [1].

(i) The Vector Spherical Wave Functions: Complete and Orthogonal Solutions of the Wave Equation.

To solve the electromagnetic scattering problem, one must address the time-harmonic vector wave equation:

$$\nabla \times \nabla \times \mathbf{E} - k^2\mathbf{E} = \mathbf{0}, \tag{1}$$

where k is the wavenumber. The time dependence, assumed to be $e^{-i\omega t}$, will be omitted in the subsequent text. After obtaining the electric field from Eq. (1), the magnetic field can be calculated using

$$\mathbf{B} = \nabla \times \mathbf{E}/i\omega. \tag{2}$$

The vector spherical wave functions

$$\begin{aligned}
&\mathbf{M}_{mn}^{(J)}(kr) = \left[i\pi_{mn}(\cos\theta)\hat{\theta} - \tau_{mn}(\cos\theta)\hat{\phi}\right] z_n^J(kr)e^{im\phi},\\
&\mathbf{N}_{mn}^{(J)}(kr) =\\
&\left[\tau_{mn}(\cos\theta)\hat{\theta} + i\pi_{mn}(\cos\theta)\hat{\phi}\right] \frac{1}{kr}\frac{d\left[(kr)z_n^J(kr)\right]}{d(kr)}e^{im\phi}\\
&+ n(n+1)P_n^m(\cos\theta)\frac{z_n^J(kr)}{kr}e^{im\phi}\hat{r},
\end{aligned} \tag{3}$$

form a complete and orthogonal set of solutions to the wave equation Eq. (1) in a homogeneous region, specifically in spherical coordinates, within a homogeneous region. Here, $\left\{\hat{r}, \hat{\theta}, \hat{\phi}\right\}$ are the unit vectors in the spherical coordinate system, and the vector spherical wave functions are interconnected through the relationship denoted by

$$\begin{aligned}
\mathbf{M}_{mn}^{(J)}(kr) =& \frac{1}{k}\nabla \times \mathbf{N}_{mn}^{(J)}(kr)\\
\mathbf{N}_{mn}^{(J)}(kr) =& \frac{1}{k}\nabla \times \mathbf{M}_{mn}^{(J)}(kr)
\end{aligned} \tag{4}$$

In Eq. (3), z_n^J represents the radial dependence and can be chosen from any of the four available functions

$$z_n^J = \begin{cases} j_n & \text{if } J = 1,\\ y_n & \text{if } J = 2,\\ h_n^{(1)} & \text{if } J = 3,\\ h_n^{(2)} & \text{if } J = 4, \end{cases} \tag{5}$$

where j_n is the spherical Bessel function, y_n is the spherical Neumann function, and $h_n^{(1)}$ and $h_n^{(2)}$ are the spherical Hankel function of the first and second kind, respectively. The angular dependent functions are defined as

$$\begin{aligned}
\pi_{mn}(\cos\theta) =& \frac{m}{\sin\theta}P_n^m(\cos\theta),\\
\tau_{mn}(\cos\theta) =& \frac{d}{d\theta}P_n^m(\cos\theta),
\end{aligned} \tag{6}$$

where

$$P_n^m(x) = \frac{1}{2^n n!}(1-x^2)^{m/2}\frac{d^{n+m}}{dx^{n+m}}\left[(x^2-1)^n\right] \tag{7}$$

is the associated Legendre function of the first kind and of degree $n = 1, 2, 3...$ and order $m = 0, \pm1, \pm2, ... \pm n$. In the context of the Mie scattering theory pertaining to a single sphere, the vector spherical wave functions (designated as Eq. (3)) are the quasi-normal modes.

(ii) Coupled Linear Equations for the Interactive Scattering Coefficients.

The Mie scattering theory pertains to the electromagnetic scattering phenomenon involving a group of isotropic spheres illuminated by a monochromatic incident wave. Let us consider a cluster comprising N spheres, each possessing dielectric constant ϵ_j and permeability μ_j, while the background medium has respective constants ϵ and μ. Within a region of uniform properties, the solutions to the wave equation in spherical coordinates are the vector spherical harmonics (3), which form a complete and orthogonal set. Consequently, any arbitrary initial incident wave can be expressed as a series of

the vector spherical wave functions.

$$\mathbf{E}_0(\mathbf{r}_j) = -\sum_{n=1}^{\infty}\sum_{m=-n}^{n} iE_{mn}\left[p_{mn}^{(j\to j)}\mathbf{N}_{mn}^{(1)}(k\mathbf{r}_j) + q_{mn}^{(j\to j)}\mathbf{M}_{mn}^{(1)}(k\mathbf{r}_j)\right], \tag{8}$$

where $\mathbf{r}_j$ is the vector pointing from the sphere's centre to the observation point,

$$E_{mn} = E_0 i^n (2n+1)\frac{(n-m)!}{(n+m)!}, \tag{9}$$

and $\left\{p_{mn}^{(j\to j)}, q_{mn}^{(j\to j)}\right\}$ represent the expansion coefficients pertaining to the initial incident wave. It is assumed that these coefficients are already known within the context of the problem being discussed.

By expressing the incident field that impinges upon the surface of the jth sphere, denoted as $\mathbf{E}_{\text{inc}}^{(j)}$, the scattered field $\mathbf{E}_{\text{sca}}^{(j)}$, and the internal field $\mathbf{E}_{\text{int}}^{(j)}$, as a set of vector spherical harmonics in the coordinate frame centered at sphere j, and subsequently applying the established standard boundary conditions

$$\begin{aligned}[\mathbf{E}_{\text{inc}}(\mathbf{r}_j) + \mathbf{E}_{\text{sca}}(\mathbf{r}_j) - \mathbf{E}_{\text{int}}(\mathbf{r}_j)] \times \hat{r}_j = \mathbf{0},\\ [\mathbf{H}_{\text{inc}}(\mathbf{r}_j) + \mathbf{H}_{\text{sca}}(\mathbf{r}_j) - \mathbf{H}_{\text{int}}(\mathbf{r}_j)] \times \hat{r}_j = \mathbf{0},\end{aligned} \tag{10}$$

over the surface of sphere, after some algebras, one arrives at

$$\mathbf{E}_{\text{inc}}^{(j)} = -\sum_{n=1}^{\infty}\sum_{m=-n}^{n} iE_{mn}\left[p_{mn}^{(j)}\mathbf{N}_{mn}^{(1)} + q_{mn}^{(j)}\mathbf{M}_{mn}^{(1)}\right], \tag{11}$$

$$\mathbf{E}_{\text{sca}}^{(j)} = \sum_{n=1}^{\infty}\sum_{m=-n}^{n} iE_{mn}\left[a_n^{(j)}p_{mn}^{(j)}\mathbf{N}_{mn}^{(3)} + b_n^{(j)}q_{mn}^{(j)}\mathbf{M}_{mn}^{(3)}\right], \tag{12}$$

$$\mathbf{E}_{\text{int}}^{(j)} = -\sum_{n=1}^{\infty}\sum_{m=-n}^{n} iE_{mn}\left[d_n^{(j)}p_{mn}^{(j)}\mathbf{N}_{mn}^{(1)} + c_n^{(j)}q_{mn}^{(j)}\mathbf{M}_{mn}^{(1)}\right], \tag{13}$$

where

$$\begin{aligned}
a_n^{(j)} =& \frac{\mu m_j^2 j_n(m_j x_j)\,[x_j j_n(x_j)]' - \mu_j j_n(x_j)\,[m_j x_j j_n(m_j x_j)]'}{\mu m_j^2 j_n(m_j x_j)\left[x_j h_n^{(1)}(x_j)\right]' - \mu_j h_n^{(1)}(x_j)\,[m_j x_j j_n(m_j x_j)]'},\\
b_n^{(j)} =& \frac{\mu_j j_n(m_j x_j)\,[x_j j_n(x_j)]' - \mu j_n(x_j)\,[m_j x_j j_n(m_j x_j)]'}{\mu_j j_n(m_j x_j)\left[x_j h_n^{(1)}(x_j)\right]' - \mu h_n^{(1)}(x_j)\,[m_j x_j j_n(m_j x_j)]'},\\
c_n^{(j)} =& \frac{\mu_j j_n(x_j)\left[x_j h_n^{(1)}(x_j)\right]' - \mu_j h_n^{(1)}(x_j)\,[x_j j_n(x_j)]'}{\mu_j j_n(m_j x_j)\left[x_j h_n^{(1)}(x_j)\right]' - \mu h_n^{(1)}(x_j)\,[m_j x_j j_n(m_j x_j)]'},\\
d_n^{(j)} =& \frac{\mu_j m_j j_n(x_j)\left[x_j h_n^{(1)}(x_j)\right]' - \mu_j m_j h_n^{(1)}(x_j)\,[x_j j_n(x_j)]'}{\mu m_j^2 j_n(m_j x_j)\left[x_j h_n^{(1)}(x_j)\right]' - \mu_j h_n^{(1)}(x_j)\,[m_j x_j j_n(m_j x_j)]'},
\end{aligned} \tag{14}$$

represent the Mie coefficients for a single sphere. The prime indicates differentiation with respect to the argument within the parentheses. The size parameter of sphere j is denoted as $x_j = k_j a_j$, where k_j represents the wavenumber and a_j represents the radius of sphere j. Additionally, m_j can be calculated as the ratio of k_j to k. Note that in our terminology, the initial incident wave (Eq. (8)) is different from the total incident wave (Eq. (11)). The only unknown quantities in Eq. (11)-(13) are $\left\{p_{mn}^{(j)}, q_{mn}^{(j)}\right\}$, which are the expansion coefficients for the total incident field striking each sphere.

By recognizing the separate components contributing to the incident field of each sphere, we can derive an alternative representation. These components include the initial incident wave, denoted as $\mathbf{E}_0$, which affects the entire cluster, and the cumulative scattered waves from the other spheres, represented as $\sum_{l\neq j}\mathbf{E}_{\text{sca}}^{(l)}$. By applying the translation addition theorem for the vector spherical harmonics (Eq. (20)), we arrive at

$$\begin{aligned}\mathbf{E}_{\text{inc}}^{(j)} &= \mathbf{E}_0 + \sum_{l\neq j}^{(l,N)}\mathbf{E}_{\text{sca}}^{(l)}\\ &= -\sum_{l=1}^{N}\sum_{n=1}^{\infty}\sum_{m=-n}^{n} iE_{mn}\left[p_{mn}^{(l\to j)}\mathbf{N}_{mn}^{(1)}(k\mathbf{r}_j) + q_{mn}^{(l\to j)}\mathbf{M}_{mn}^{(1)}(k\mathbf{r}_j)\right],\end{aligned} \tag{15}$$

where

$$p_{mn}^{(l\to j)} = -\sum_{v=1}^{\infty}\sum_{u=-v}^{v}\left[A_{mn}^{uv(3)}(l\to j)a_v^{(l)}p_{uv}^{(l)} + B_{mn}^{uv(3)}(l\to j)b_v^{(l)}q_{uv}^{(l)}\right], \tag{16}$$

$$q_{mn}^{(l\to j)} = -\sum_{v=1}^{\infty}\sum_{u=-v}^{v}\left[B_{mn}^{uv(3)}(l\to j)a_v^{(l)}p_{uv}^{(l)} + A_{mn}^{uv(3)}(l\to j)b_v^{(l)}q_{uv}^{(l)}\right], \tag{17}$$

for $l \neq j$ denote the scattered wave of a sphere l, expanded in a coordinate system centered at sphere j. The coordinate axes of both systems are parallel to each other. And $\left\{A_{mn}^{uv(3)}(l\to j), B_{mn}^{uv(3)}(l\to j)\right\}$ are the normalized translation coefficients defined in Eq. (26).

By setting Eq. (11) equal to Eq. (15) and utilizing Eq. (16-17) along with the orthogonal properties of vector spherical harmonics, we obtain a system of linear equations governing the interactive scattering coefficients

$$\begin{aligned} p_{mn}^{(j)} &= \sum_{l=1}^{N} p_{mn}^{(l\to j)} = p_{mn}^{(j\to j)} - \sum_{l\neq j}^{(1,N)}\sum_{v=1}^{\infty}\sum_{u=-v}^{v}\left[A_{mn}^{uv(3)}(l\to j)a_v^{(l)}p_{uv}^{(l)} + B_{mn}^{uv(3)}(l\to j)b_v^{(l)}q_{uv}^{(l)}\right], \\ q_{mn}^{(j)} &= \sum_{l=1}^{N} q_{mn}^{(l\to j)} = q_{mn}^{(j\to j)} - \sum_{l\neq j}^{(1,N)}\sum_{v=1}^{\infty}\sum_{u=-v}^{v}\left[B_{mn}^{uv(3)}(l\to j)a_v^{(l)}p_{uv}^{(l)} + A_{mn}^{uv(3)}(l\to j)b_v^{(l)}q_{uv}^{(l)}\right]. \end{aligned} \tag{18}$$

Equation Eq. (18) is the main equation for the multiple scattering theory.

The sum of the angular momentum index v in Eq. (18) can be limited to a finite value $L_{\max}$ [1]. This is indicated by the observation that the individual sphere Mie coefficients $a_n^{(l)}$ tend to zero as n becomes much larger than x_l. Thus

$$\begin{aligned} p_{mn}^{(j)} &= p_{mn}^{(j\to j)} - \sum_{l\neq j}^{(1,N)}\sum_{v=1}^{L_{\max}}\sum_{u=-v}^{v}\left[A_{mn}^{uv(3)}(l\to j)a_v^{(l)}p_{uv}^{(l)} + B_{mn}^{uv(3)}(l\to j)b_v^{(l)}q_{uv}^{(l)}\right], \\ q_{mn}^{(j)} &= q_{mn}^{(j\to j)} - \sum_{l\neq j}^{(1,N)}\sum_{v=1}^{L_{\max}}\sum_{u=-v}^{v}\left[B_{mn}^{uv(3)}(l\to j)a_v^{(l)}p_{uv}^{(l)} + A_{mn}^{uv(3)}(l\to j)b_v^{(l)}q_{uv}^{(l)}\right]. \end{aligned} \tag{19}$$

Equation (19) represents a system of $2NL_{\max}(L_{\max}+2)$ linear equations. This system can be solved numerically by iterative algorithms like the conjugate gradient method or the GMRES. Typically, in our calculations, we can achieve convergence by selecting $L_{\max}$ as the smallest integer greater than $kr_s + 4.05(kr_s)^{1/3} + 2$ [1], where r_s is the sphere radius. In certain exceptional cases, when the interactions between the spheres are particularly strong, a higher value of $L_{\max}$ is necessary. This is especially true when resonances are triggered.

(iii) Translation coefficients for vector spherical wave functions.

Matching the boundary conditions across the surface of each sphere is a vital aspect of the multiple-scattering theory. This objective was successfully achieved through the utilization of the translation addition theorem of the vector spherical harmonics:

$$\begin{aligned} \mathbf{M}_{uv}^{(J)}(k\mathbf{r}_l) &= \sum_{n=0}^{\infty}\sum_{m=-n}^{n}\left[\tilde{A}_{mn}^{uv(J)}(l\to j)\mathbf{M}_{mn}^{(1)}(k\mathbf{r}_j) + \tilde{B}_{mn}^{uv(J)}(l\to j)\mathbf{N}_{mn}^{(1)}(k\mathbf{r}_j)\right], \\ \mathbf{N}_{uv}^{(J)}(k\mathbf{r}_l) &= \sum_{n=0}^{\infty}\sum_{m=-n}^{n}\left[\tilde{B}_{mn}^{uv(J)}(l\to j)\mathbf{M}_{mn}^{(1)}(k\mathbf{r}_j) + \tilde{A}_{mn}^{uv(J)}(l\to j)\mathbf{N}_{mn}^{(1)}(k\mathbf{r}_j)\right], \end{aligned} \tag{20}$$

for $r < d_{ij}$ and

$$\begin{aligned}\mathbf{M}_{uv}^{(J)}(k\mathbf{r}_l) &= \sum_{n=0}^{\infty}\sum_{m=-n}^{n}\left[\tilde{A}_{mn}^{uv(1)}(l\to j)\mathbf{M}_{mn}^{(J)}(k\mathbf{r}_j) + \tilde{B}_{mn}^{uv(1)}(l\to j)\mathbf{N}_{mn}^{(J)}(k\mathbf{r}_j)\right],\\ \mathbf{N}_{uv}^{(J)}(k\mathbf{r}_l) &= \sum_{n=0}^{\infty}\sum_{m=-n}^{n}\left[\tilde{B}_{mn}^{uv(1)}(l\to j)\mathbf{M}_{mn}^{(J)}(k\mathbf{r}_j) + \tilde{A}_{mn}^{uv(1)}(l\to j)\mathbf{N}_{mn}^{(J)}(k\mathbf{r}_j)\right],\end{aligned} \tag{21}$$

for $r > d_{ij}$ where $\tilde{A}_{mn}^{uv(J)}(l \to j)$ and $\tilde{B}_{mn}^{uv(J)}(l \to j)$ represent the translation coefficients used for the conversion between the coordinate systems of sphere l and sphere j, where the axes of both systems are parallel to each other and centered at the origin. Their expressions are

$$\begin{aligned}\tilde{A}_{mn}^{uv(J)}(l\to j) = (-1)^u i^{v-n}\frac{2v+1}{2v(v+1)}\sum_{p=|n-v|}^{n+v}(-i)^p[n(n+1)+v(v+1)-p(p+1)]\\ \times a(m,n,-u,v,p) z_p^J(kd_{lj})P_p^{m-u}(\cos\theta_{lj})e^{i(m-u)\phi_{lj}},\end{aligned} \tag{22}$$

$$\begin{aligned}\tilde{B}_{mn}^{uv(J)}(l\to j) = (-1)^u i^{v-n}\frac{2v+1}{2v(v+1)}\sum_{p=|n-v|}^{n+v}(-i)^p b(m,n,-u,v,p,p-1)\\ \times z_p^J(kd_{lj})P_p^{m-u}(\cos\theta_{lj})e^{i(m-u)\phi_{lj}},\end{aligned} \tag{23}$$

where $\{d_{lj}, \theta_{lj}, \phi_{lj}\}$ are the spherical coordinates of the vector pointing from the center of sphere l to that of the sphere j and

$$a(m,n,u,v,p) = \frac{2p+1}{2}\frac{(p-m-u)!}{(p+m+u)!}\int_{-1}^{1}P_n^m(x)P_v^u(x)P_p^{m+u}(x)\,dx, \tag{24}$$

$$b(m,n,-u,v,p,p-1) = \frac{2p+1}{2p-1}\begin{bmatrix}(v-u)(v+u+1)\times a(m,n,-u-1,v,p-1)\\ -(p-m+u)(p-m+u-1)\times a(m,n,-u+1,v,p-1)\\ +2u(p-m+u)\times a(m,n,-u,v,p-1)\end{bmatrix}, \tag{25}$$

For convenience, the normalized translation coefficients are defined to be

$$\begin{aligned}A_{mn}^{uv(J)}(l\to j) &= \left(\frac{E_{uv}}{E_{mn}}\right)\tilde{A}_{mn}^{uv(J)}(l\to j),\\ B_{mn}^{uv(J)}(l\to j) &= \left(\frac{E_{uv}}{E_{mn}}\right)\tilde{B}_{mn}^{uv(J)}(l\to j).\end{aligned} \tag{26}$$

The process of deriving the translation addition theorem can be quite lengthy, so interested readers are encouraged to refer to references [2–4] for a detailed explanation. When it comes to implementing the theorem numerically, it is often more efficient to evaluate the translation coefficients using recursive algorithms [4] instead of directly applying the formulas mentioned earlier. Additionally, the technique of "symmetry and rotational decomposition" discussed in reference [5] proves to be highly beneficial as well.

QUANTUM PROCESSOR

Parameters.—The demonstration of the non-Hermitian dynamics of the given optical manipulation problem is per-

a

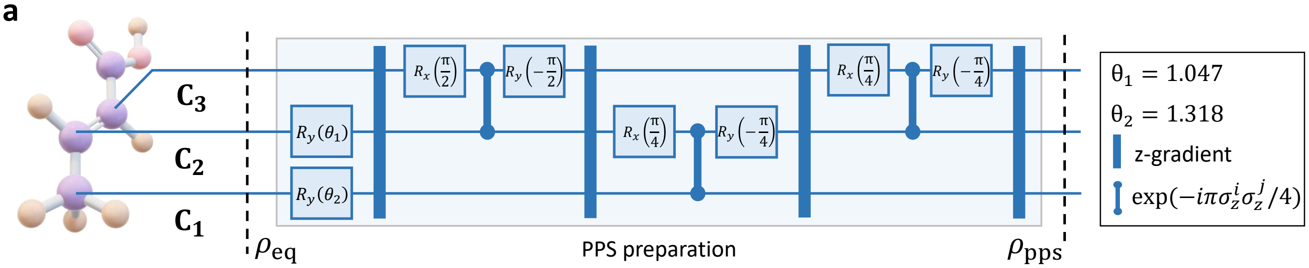

b

(Hz)	C_1	C_2	C_3
C_1	-2572.50		
C_2	41.6	-21924.48	
C_3	1.5	69.6	-18476.83
T_2 (s)	1.214	1.073	1.077

c **d**

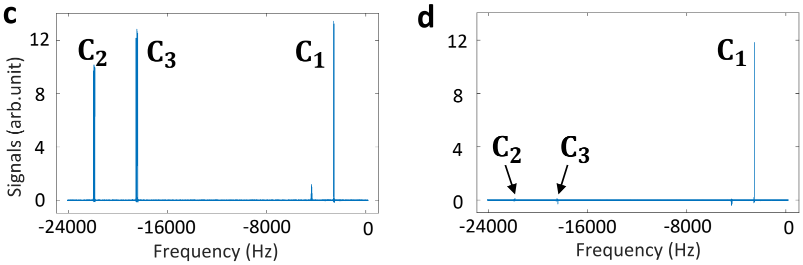

Figure S1. **Molecular structure of the sample and PPS initialization of the experiments.** **a**, Quantum circuit of the PPS preparation for crotonic acid. **b**, Parameters of the crotonic acid sample. The diagonal terms are chemical shifts, and the off-diagonal terms are the J-coupling strengths between different ^{13}C spins. The T_2 relaxation times are listed in the bottom row. **c**, Spectrum of the thermal equilibrium state. **d**, Spectrum of the PPS state, where only a single peak on C_1 is observed.

formed on the NMR spin simulator. The sample we use is the ^{13}C-labeled crotonic acid dissolved in acetone-d6. The four different ^{13}C spins have different chemical environments and we choose three of them as the three qubits for the single-particle optical trapping experiment. Each qubit can be individually controlled by radio-frequency (r.f.) pulses. All experiments are carried out on a Bruker AVANCE 600 MHz spectrometer at room temperature.

In Fig. S1a, we show the molecular structure of the crotonic acid. The three ^{13}C spins are labeled by C_1, C_2, and C_3. Under the strong magnetic field (14.1 T), the chemical shifts of these spins are distinct, enabling the implementation of arbitrary single-qubit rotations by shaped r.f. pulses. The natural interactions between the spins permits two-qubit entangling gates. In Fig. S1b, we list the chemical shifts and the J-coupling interactions between the three qubits. In the rotating frame, the internal Hamiltonian can be written as

$$\mathcal{H}_{int} = -\pi \sum_i (\nu_i - \nu_0)\sigma_z^i + \pi \sum_{j<k} \frac{J_{jk}}{2} \sigma_z^j \sigma_z^k, \tag{27}$$

where ν_i is the chemical shift of the i-th spin and ν_0 is the reference frequency of the rotating frame. Here, $i = 1, 2, 3$ refer to the three used ^{13}C spins.

We can apply the r.f. pulses to perform arbitrary single-qubit rotations. The Hamiltonian of the control field is

$$\mathcal{H}_c = \sum_i \pi B_i (\cos\phi_i \sigma_x^i + \sin\phi_i \sigma_y^i), \tag{28}$$

where we can change the amplitudes B_i and phases ϕ_i of the pulse to realize arbitrary rotations on the i-th qubit. Two-qubit gates are implemented via free evolution where the unwanted qubits are decoupled from the target qubits. For most of the pulses in our experiment, we can apply the optimal control technique, which combines the r.f. pulses and free evolutions of the internal Hamiltonian, to optimize a high-fidelity shaped pulse that can realize the target evolution.

Initialization.—At room temperature, our system stabilizes into a thermal equilibrium described by a mixed state ρ_{eq}. The thermal spectrum of the crotonic acid sample is displayed in Fig. S1c. In NMR quantum computation, instead of realizing a genuine pure state, we initialize the system into a pseudo-pure state (PPS), which is a combination of a pure state with a large identity component. This initialization involves non-unitary operations. In the following, we show the quantum circuit to prepare the PPS from the thermal equilibrium.

Our experiments, conducted at ambient conditions, yield a thermal state for the spin system, which can be succinctly described by

$$\rho_{\text{eq}} = \frac{\mathbb{I}}{2^3} + \epsilon \sum_{i=1}^{3} \sigma_z^i, \tag{29}$$

where $\mathbb{I}$ is the 8×8 identity matrix, and ϵ, the polarization factor, is on the order of 10^{-5}. This thermal state is derived from the Boltzmann distribution, formalized as

$$\rho_{\text{eq}} = \frac{\exp(-\beta \mathcal{H}^{\text{lab}})}{\text{tr}[\exp(-\beta \mathcal{H}^{\text{lab}})]}, \tag{30}$$

with $\mathcal{H}^{\text{lab}}$ representing the system Hamiltonian in the laboratory frame, defined as $-\pi \sum_i \nu_i^{\text{lab}} \sigma_z$, and β denoting the inverse temperature $1/kT$. It is important to note that this

Hamiltonian differs from $\mathcal{H}_{int}$ in the rotating frame. Given that kT is much greater than any Hamiltonian eigenvalue E_n at room temperature, we approximate $\exp(-\beta H)$ by $\mathbb{I} - \beta H$, simplifying our expression to

$$\rho_{\text{eq}} = \frac{\mathbb{I}}{8} - \frac{\beta}{8}\mathcal{H}^{\text{lab}}. \tag{31}$$

In the laboratory frame, the Larmor frequencies ν_i^{lab}, which are in the MHz range and significantly exceed the coupling strengths J_{ij}, are the primary contributors to the thermal state. These frequencies are approximately $\gamma_C B_0/2\pi$, leading to:

$$\rho_{\text{eq}} = \frac{\mathbb{I}}{8} + \frac{\beta\gamma_C B_0}{16}\sum_{i=1}^{3}\sigma_z^i, \tag{32}$$

where γ_C is the gyromagnetic ratio of ^{13}C, and B_0 is the static magnetic field strength. This approximation is valid as the Larmor frequencies for all ^{13}C spins are nearly identical, despite chemical shift variations. By setting $\epsilon = \beta\gamma_C B_0/16$, we achieve the desired thermal equilibrium state in Eq. (29).

The initial state of the NMR system is established using the spatial averaging technique. This method utilizes a specific pulse sequence, which is illustrated in Fig. S1a. For simplicity, we denote the equilibrium state density matrix as $\rho_{\text{eq}} = \text{ZII} + \text{IZI} + \text{IIZ}$.

The system undergoes two initial rotation operations, $R_y(\theta_1)$ and $R_y(\theta_2)$, where $\theta_1 = \arccos(0.5)$ and $\theta_2 = \arccos(0.25)$. These rotations transform the system into the state:

$$\frac{1}{4}\text{ZII} + \frac{\sqrt{15}}{4}\text{XII} + \frac{1}{2}\text{IZI} + \frac{\sqrt{3}}{2}\text{IXI} + \text{IIZ}.$$

Subsequently, a z-gradient field is applied to dephase the spins, resulting in the state:

$$\frac{1}{4}\text{ZII} + \frac{1}{2}\text{IZI} + \text{IIZ}.$$

To generate the IZZ element, three operations are performed: 1. A rotation $R_x(\frac{\pi}{2})$ on C_3 yields:

$$\frac{1}{4}\text{ZII} + \frac{1}{2}\text{IZI} - \text{IIY}.$$

2. An operation $e^{-i\pi\sigma_z^2\sigma_z^3/4}$ on C_2 and C_3 changes the system to:

$$\frac{1}{4}\text{ZII} + \frac{1}{2}\text{IZI} + \text{IZX}.$$

3. A rotation $R_y(-\frac{\pi}{2})$ on C_3 results in:

$$\frac{1}{4}\text{ZII} + \frac{1}{2}\text{IZI} + \text{IZZ}.$$

Another z-gradient field is applied to minimize errors.

Four additional operations are executed to create the ZZI and ZZZ element: 1. A rotation $R_x(\frac{\pi}{4})$ on C_2 modifies the system to:

$$\frac{1}{4}\text{ZII} + \frac{\sqrt{2}}{4}\text{IZI} - \frac{\sqrt{2}}{4}\text{IYI} + \frac{\sqrt{2}}{2}\text{IZZ} - \frac{\sqrt{2}}{2}\text{IYZ}.$$

2. An operation $e^{-i\pi\sigma_z^1\sigma_z^2/4}$ on C_1 and C_2 transforms the system into:

$$\frac{1}{4}\text{ZII} + \frac{\sqrt{2}}{4}\text{IZI} + \frac{\sqrt{2}}{4}\text{ZXI} + \frac{\sqrt{2}}{2}\text{IZZ} + \frac{\sqrt{2}}{2}\text{ZXZ}.$$

3. A rotation $R_y(-\frac{\pi}{4})$ on C_2 leads to:

$$\frac{1}{4}\text{ZII}+\frac{1}{4}\text{IZI}-\frac{1}{4}\text{IXI}+\frac{1}{4}\text{ZXI}+\frac{1}{4}\text{ZZI}+\frac{1}{2}\text{IZZ}-\frac{1}{2}\text{IXZ}+\frac{1}{2}\text{ZXZ}+\frac{1}{2}\text{ZZZ}.$$

4. After a z-gradient field application, the system becomes:

$$\frac{1}{4}\text{ZII} + \frac{1}{4}\text{IZI} + \frac{1}{4}\text{ZZI} + \frac{1}{2}\text{IZZ} + \frac{1}{2}\text{ZZZ}.$$

The final set of four operations aims to create the IIZ and ZIZ element: 1. A rotation $R_x(\frac{\pi}{4})$ on C_3 adjusts the system to:

$$\frac{1}{4}\text{ZII}+\frac{1}{4}\text{IZI}+\frac{1}{4}\text{ZZI}+\frac{\sqrt{2}}{4}\text{IZZ}-\frac{\sqrt{2}}{4}\text{IZY}+\frac{\sqrt{2}}{4}\text{ZZZ}-\frac{\sqrt{2}}{4}\text{ZZY}.$$

2. An operation $e^{-i\pi\sigma_z^1\sigma_z^2/4}$ on C_2 and C_3 modifies the system to:

$$\frac{1}{4}\text{ZII}+\frac{1}{4}\text{IZI}+\frac{1}{4}\text{ZZI}+\frac{\sqrt{2}}{4}\text{IZZ}+\frac{\sqrt{2}}{4}\text{IIX}+\frac{\sqrt{2}}{4}\text{ZZZ}+\frac{\sqrt{2}}{4}\text{ZIX}.$$

3. A rotation $R_y(-\frac{\pi}{4})$ on C_3 results in:

$$\frac{1}{4}\text{ZII} + \frac{1}{4}\text{IZI} + \frac{1}{4}\text{ZZI} + \frac{1}{4}\text{IZZ} - \frac{1}{4}\text{IZX} + \frac{1}{4}\text{IIX} + \frac{1}{4}\text{IIZ} + \frac{1}{4}\text{ZZZ} - \frac{1}{4}\text{ZZX} + \frac{1}{4}\text{ZIX} + \frac{1}{4}\text{ZIZ}.$$

4. Following a z-gradient field, the system attains the final state:

$$\frac{1}{4}\text{ZII} + \frac{1}{4}\text{IZI} + \frac{1}{4}\text{ZZI} + \frac{1}{4}\text{IZZ} + \frac{1}{4}\text{IIZ} + \frac{1}{4}\text{ZZZ} + \frac{1}{4}\text{ZIZ}.$$

The resulting density matrix represents the desired PPS:

$$\rho_{\text{PPS}} = \frac{1-\epsilon}{8}\mathbb{I} + \epsilon|000\rangle\langle 000|. \tag{33}$$

Since the identity matrix component is invariant under any unitary transformation and does not affect the NMR signal, the quantum system behaves as if it were in the pure state $|000\rangle\langle 000|$, scaled by a factor.

Readout.—We use quantum state tomography (QST) to get the final states after different evolutions. In the NMR system, we can read out the first-order coherent terms (only one spin in X or Y while the other two spins in Z or I) for a given spin in a single experiment. For other Pauli terms, we can apply $\pi/2$ pulses to rotate them into the first-order coherent states. For our experiment, there are in total five readout pulses: $\text{I}, R_y^2(\pi/2), R_x^2(\pi/2), R_y^3(\pi/2), R_x^1(\pi/2)$. Compatible QST can be realized after this readout stage.

SINGLE-PARTICLE EXPERIMENT

Firstly, let us briefly review the general approach of simulating the non-Hermitian optical trapping model using a quantum computer. Directly loading the classical force matrix into a quantum computer is not straightforward, due to the requirement for normalized vectors and preference of unitary matrices on quantum computers. As a result, both vectors that encode displacements and velocities should be transformed accordingly. Considering the classical equation $\ddot{\mathbf{X}} - \tilde{\mathbf{K}}\mathbf{X} = 0$, we define $\mathbf{Z}_1 = \mathbf{X}$, $\mathbf{Z}_2 = \dot{\mathbf{X}}$, and $\mathbf{Z} = \begin{bmatrix} \mathbf{Z}_1 \\ \mathbf{Z}_2 \end{bmatrix}$. The equation is reformulated as

$$\begin{cases} \dot{\mathbf{Z}}_1 = \mathbf{Z}_2 \\ \dot{\mathbf{Z}}_2 = \tilde{\mathbf{K}}\mathbf{Z}_1 \end{cases} \quad \text{or} \quad \dot{\mathbf{Z}} = \begin{bmatrix} \tilde{\mathbf{0}} & \tilde{\mathbf{I}} \\ \tilde{\mathbf{K}} & \tilde{\mathbf{0}} \end{bmatrix} \mathbf{Z} = \tilde{\mathbf{K}}'\mathbf{Z}, \tag{34}$$

where $\tilde{\mathbf{I}}$ denotes the identity matrix that has the same dimension as that of $\tilde{\mathbf{K}}$. The solution to this equation is straightforwardly given by:

$$\mathbf{Z}(t) = e^{\tilde{\mathbf{K}}'t}\mathbf{Z}(0). \tag{35}$$

The initial vector $\mathbf{Z}(0)$ can be normalized and encoded by a pure quantum state. However, $e^{\tilde{\mathbf{K}}'t}$ is typically not a unitary matrix, and solving for this matrix becomes challenging as the system's dimension increases. To implement this operation on a quantum computer, we resort to a Taylor expansion and retain the first two terms to iteratively determine the trajectory. In most instances, neither $\tilde{\mathbf{K}}'$ nor $\tilde{\mathbf{K}}'^2$ is unitary. To address this, we introduce ancillary qubits. Since any matrix can be expressed as a linear combination of unitary matrices, we assume $\tilde{\mathbf{K}}'^m = \sum_{i=1}^n c_i^m \tilde{\mathbf{U}}_i^m$, and with no more than $k = \log_2 l$ ancillary qubits, we can perform a non-unitary operation akin to $\tilde{\mathbf{K}}'$ on a quantum state. This part has been described in the main text.

For the single-particle case, both $\tilde{\mathbf{K}}'$ and $\tilde{\mathbf{K}}'^2$ can be divided into two unitary matrices:

$$\tilde{\mathbf{K}}'^2 = \begin{bmatrix} a+b & g & & \\ -g & a-b & & \\ & & a+b & g \\ & & -g & a-b \end{bmatrix} = \epsilon \frac{1}{\epsilon} \begin{bmatrix} a & g & & \\ -g & a & & \\ & & a & g \\ & & -g & a \end{bmatrix} + b \begin{bmatrix} 1 & 0 & & \\ 0 & -1 & & \\ & & 1 & 0 \\ & & 0 & -1 \end{bmatrix}, \tag{36}$$

$$\tilde{\mathbf{K}}' = \begin{bmatrix} & & 1 & 0 \\ & & 0 & 1 \\ a+b & g & & \\ -g & a-b & & \end{bmatrix} \to \epsilon \frac{1}{\epsilon} \begin{bmatrix} & & \epsilon & 0 \\ & & 0 & \epsilon \\ a & g & & \\ -g & a & & \end{bmatrix} + b \begin{bmatrix} & & 1 & 0 \\ & & 0 & 1 \\ 1 & 0 & & \\ 0 & 1 & & \end{bmatrix}, \tag{37}$$

where $\epsilon = \sqrt{a^2 + g^2}$. Hence, we have decomposed $\tilde{\mathbf{K}}'$ and $\tilde{\mathbf{K}}'^2$ into the linear combination of two unitaries: $\tilde{\mathbf{K}}' = c_1^1\tilde{\mathbf{U}}_1^1 + c_2^1\tilde{\mathbf{U}}_1^1$, $\tilde{\mathbf{K}}'^2 = c_1^2\tilde{\mathbf{U}}_1^2 + c_2^2\tilde{\mathbf{U}}_2^2$.

The quantum circuit for the single-particle case is depicted in Fig. S2. While we have discussed about how to realize the second-order Taylor expansion term in the main text, the subsequent discussion only relies on the realization of the first-order term $\tilde{\mathbf{K}}'\mathbf{Z}(0)$. The initial qubit serves as the ancillary qubit. The unitary operation $\tilde{\mathbf{V}}$ prepares this qubit into the quantum state $\frac{1}{\sqrt{c_1^1+c_2^1}}[\sqrt{c_1^1}, \sqrt{c_2^1}]^T$. The subsequent two qubits, which span a 4-dimensional space, encode the information of the vector $\mathbf{Z}$. The operation $\tilde{\mathbf{U}}_0$ is a two-qubit gate that initializes the last two qubits into the normalized quantum state $\frac{\mathbf{Z}(0)}{\|\mathbf{Z}(0)\|}$. Therefore, the input quantum state of the entire system is represented as

$$|\Psi_1\rangle = \frac{1}{\sqrt{c_1^1 + c_2^1}} \begin{bmatrix} \sqrt{c_1^1} \\ \sqrt{c_2^1} \end{bmatrix} \otimes \frac{\mathbf{Z}(0)}{\|\mathbf{Z}(0)\|}. \tag{38}$$

Following are the controlled operations between the ancillary qubit and system qubits. The first controlled operation applies the unitary $\tilde{\mathbf{U}}_1^1$ to the system qubits only if the ancillary qubit is in the state $|0\rangle$. Conversely, the unitary $\tilde{\mathbf{U}}_2^1$ is applied under the same conditions but when the ancillary qubit is in the state $|1\rangle$. This delineates the ancillary qubit's role: to selectively employ $\tilde{\mathbf{U}}_1^1$ and $\tilde{\mathbf{U}}_2^1$ in a ratio that effectively applies the operation $c_1^1\tilde{\mathbf{U}}_1^1 + c_2^1\tilde{\mathbf{U}}_2^1$ (scaled by a constant). After the controlled operations, the quantum state evolves to

$$|\Psi_2\rangle = \frac{\sqrt{c_1^1}|0\rangle}{\sqrt{c_1^1 + c_2^1}} \otimes \frac{\tilde{\mathbf{U}}_1^1\mathbf{Z}(0)}{\|\mathbf{Z}(0)\|} + \frac{\sqrt{c_2^1}|1\rangle}{\sqrt{c_1^1 + c_2^1}} \otimes \frac{\tilde{\mathbf{U}}_2^1\mathbf{Z}(0)}{\|\mathbf{Z}(0)\|}. \tag{39}$$

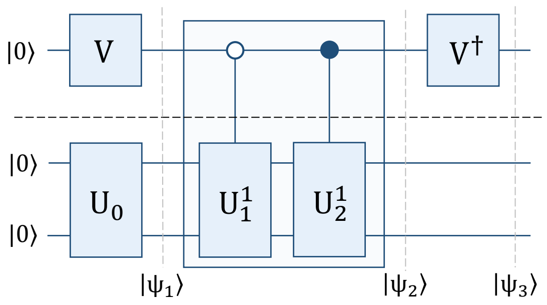

Figure S2. **Quantum circuit for the single-particle OT experiment.** $\tilde{\mathbf{U}}_0$ is the unitary operator to load the initial states (displacements and velocities) of particle into the system qubits. $\tilde{\mathbf{V}}$ is to prepare the ancillary qubit to $\frac{1}{\sqrt{c_1^1+c_2^1}}[\sqrt{c_1^1},\sqrt{c_2^1}]^T$. The following controlled gates are to equivalently apply $\tilde{\mathbf{K}}' = c_1^1\tilde{\mathbf{U}}_1^1 + c_2^1\tilde{\mathbf{U}}_1^1$ to the system qubit. $\tilde{\mathbf{V}}^\dagger$ is to rotate the ancillary qubit back to $|0\rangle$.

The operator $\tilde{\mathbf{V}}^\dagger$, the Hermitian adjoint of $\tilde{\mathbf{V}}$, rotates the ancillary qubit back to the state $|0\rangle$, transitioning the system into the state

$$|\Psi_3\rangle = |0\rangle \otimes \frac{c_1^1\tilde{\mathbf{U}}_1^1 + c_2^1\tilde{\mathbf{U}}_1^1}{c_1^1 + c_2^1}\frac{\mathbf{Z}(0)}{\|\mathbf{Z}(0)\|}. \tag{40}$$

Thus, by measuring only the last two qubits when the ancillary qubit is in the state $|0\rangle$, we can extract the result of $(c_1^1\tilde{\mathbf{U}}_1^1+c_2^1\tilde{\mathbf{U}}_2^1)\mathbf{Z}(0)$, which corresponds to $\tilde{\mathbf{K}}'\mathbf{Z}(0)$ multiplied by a constant that can be easily accounted for.

EXPERIMENTAL RE-NORMALIZATION

Re-normalization of the classical vectors.—As delineated in the main text, classical vectors cannot be directly encoded onto system qubits. To initialize the quantum system, the classical vector must first be normalized to conform to quantum computing requirement. Yet, for contemporary quantum computers with significant noise, normalization alone is insufficient. The predefined conditions of our scenario reveal that during the dynamic process, displacements are approximately 200 times greater than velocities. On a quantum computer, all terms of quantum vectors are processed uniformly, which implies that errors are likely to be of comparable magnitude across all terms. Given that displacement terms are two orders of magnitude larger than velocity terms, the relative error for velocity terms is magnified, potentially resulting in erratic trajectories.

The remedy is straightforward: "re-normalize" the vector to manually balance the magnitudes of the two term types. This "re-normalization" ensures that the measured quantities fall within an optimal range for quantum measurement. With displacement terms being about 200 times larger than velocity terms, we scale only the velocity terms by a factor of 200 and then re-normalize the vector. This procedure is reversible due to the structure of the matrix $\tilde{\mathbf{K}}'$ (and $\tilde{\mathbf{K}}'^2$), which is anti-block (block) diagonalized, allowing us to scale only the displacement (velocity) terms and adjust the results by the normalization and re-normalization factors to retrieve precise outcomes.

The efficacy of this approach is shown in Fig. S3. Figure S3a illustrates the populations of the vector's four terms without "re-normalization," whereas Fig. S3b demonstrates the method's application. In Fig. S3b, the term populations are notably more balanced, and the states are more distinguishable. The experimental results, presented in Figs. S3c and d, validate that employing this technique mitigates error amplification.

Re-balance of the LCU coefficients.— In the matrix $\tilde{\mathbf{K}}'$ (see Eq. (37)), one may observe that the upper right element is set to 1 rather than $\epsilon + b$. While this is accurate, it does not impact the data processing since each displacement term is consistently scaled by this coefficient during matrix multiplication. To ensure accurate data extraction from the quantum computer at each step, the coefficient $1/(\epsilon + b)$ must be applied to the displacement terms, guaranteeing the input vector is correctly engaged with $\tilde{\mathbf{K}}'$.

Furthermore, this step is beneficial for achieving more reliable simulation outcomes on contemporary quantum computers, which are subject to significant noise. Neglecting this step and attempting to use an additional ancillary qubit to implement the original matrix is also feasible. As an example, matrix $\tilde{\mathbf{K}}'$ can also be divided strictly with three unitary matrices $\tilde{\mathbf{K}}' = c_{31}\tilde{\mathbf{U}}_{31} + c_{32}\tilde{\mathbf{U}}_{32} + c_{33}\tilde{\mathbf{U}}_{33}$:

$$\tilde{\mathbf{K}}' = \begin{bmatrix} & & 1 & 0 \\ & & 0 & 1 \\ a+b & g & & \\ -g & a-b & & \end{bmatrix} = \epsilon\frac{1}{\epsilon}\begin{bmatrix} & & \epsilon & 0 \\ & & 0 & \epsilon \\ a & g & & \\ -g & a & & \end{bmatrix} + \frac{(1-\epsilon+b)}{2}\begin{bmatrix} & & 1 & 0 \\ & & 0 & 1 \\ 1 & 0 & & \\ 0 & 1 & & \end{bmatrix} + \frac{(1-\epsilon-b)}{2}\begin{bmatrix} & & 1 & 0 \\ & & 0 & 1 \\ -1 & 0 & & \\ 0 & -1 & & \end{bmatrix}, \tag{41}$$

where $c_{31} = \epsilon$, $c_{32} = (1-\epsilon+b)/2$, and $c_{33} = (1-\epsilon-b)/2$.

However, this distribution way of LCU coefficients would

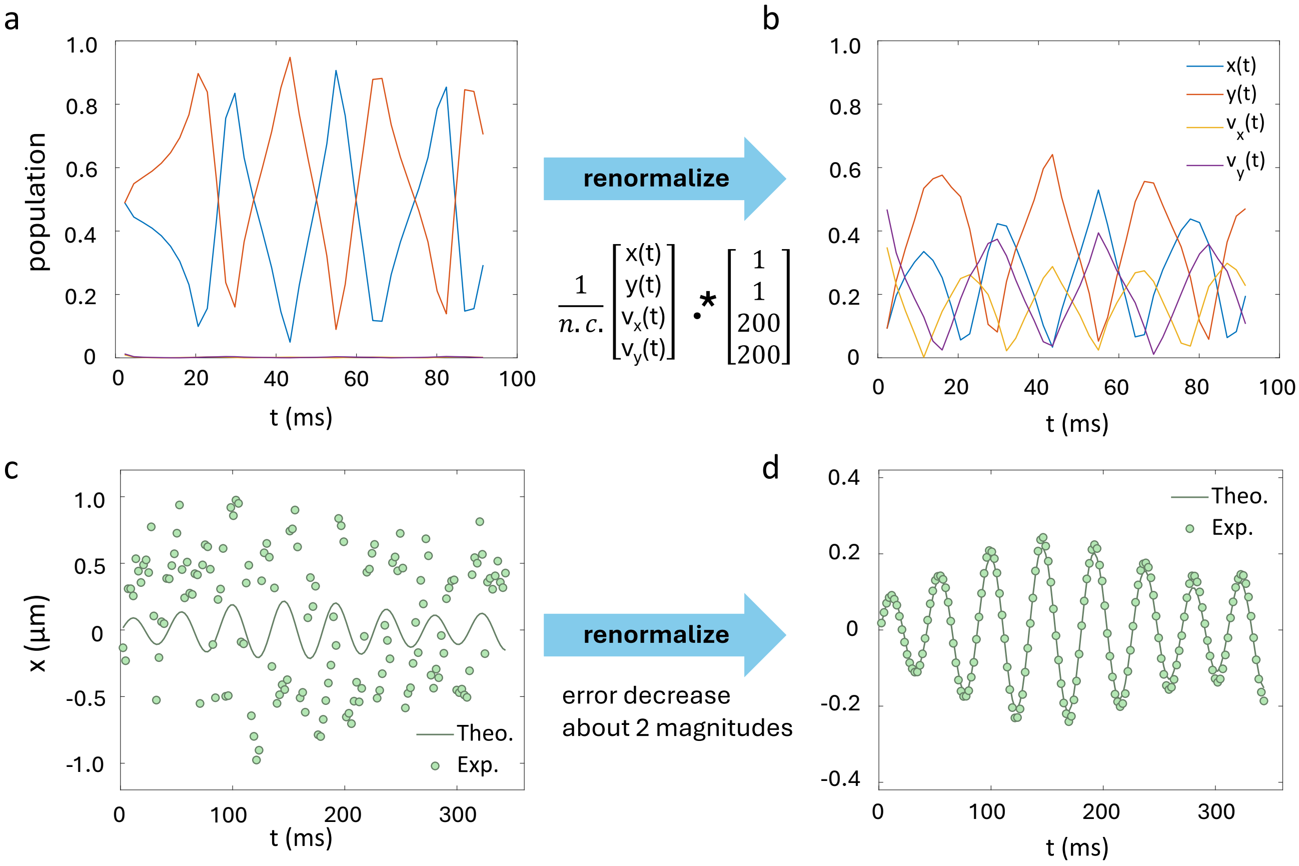

Figure S3. **Re-normalization of the states.** **a**, The amplitudes of the four terms (displacements and velocities in the x-y plane) of the vector without re-normalization. Compared with displacements, the velocities are much smaller, making it difficult to be accurately loaded into quantum states. **b**, After re-normalization, the amplitudes of the four terms are much more balanced. **c, d**, Experimental results without and with employing re-normalization.

result in a substantial disparity between the magnitudes of c_{31} between c_{32} and c_{33} (1 is much greater than ϵ, as a comparison, in Eq. (37), the two LCU coefficients are ϵ and b, which roughly maintain in the same magnitude). This discrepancy would cause the coefficients c_i to vary greatly in size, complicating the precise preparation of the ancillary qubits. The employed method maintains the coefficients c_i within a comparable range, significantly reducing potential errors in preparing the ancillary qubits for the desired states and enhancing the accuracy of the reconstructed trajectories. This "re-balanc" step balance the coefficients to make they more adaptive to a quantum computer.

Extraction of experimental results.— After the above re-normalization step, we need to post-process the experimental results correspondingly. At the end of the quantum circuit, the information from each step is encapsulated in a final 8×8 density matrix. We are only interested in the system qubits (excluding the ancillary part). To extract the relevant information, we first apply QST to the system qubits, resulting in a 4×4 matrix. In the ideal case, this matrix should represent a pure state containing the desired vector. In experiment, this matrix becomes a mixed state due to the noise, so we employ maximum likelihood estimation to extract the closest pure state as the outcome. This pure state represents the experimentally measured vector of first-order Taylor expansion $\tilde{\mathbf{K}}'\mathbf{Z}(0)$ or second-order Taylor expansion $\tilde{\mathbf{K}}'^2\mathbf{Z}(0)$. For either one, we multiply the vector by $b+\epsilon$ as required by the form of $\tilde{\mathbf{K}}'$ and $\tilde{\mathbf{K}}'^2$.

Subsequently, when dealing with the first-order Taylor expansion term, we multiply the the displacement terms (the first two terms) of the vector $\mathbf{Z}(t)$ by $1/(b+\epsilon)$, effectively reversing the influence of the "re-balance" step. Conversely, when handling the second-order Taylor expansion term, no adjustments are made. This step ensures that the measured quantities remain within an appropriate range. To reverse the "re-normalization" step, we multiply the vector by the re-normalization factor and by $1/200$ for displacement (velocity) terms in the first-order (second-order) terms.

Experimental error analysis.— Here, we present the fidelity of the measured final states for each of the three situations in

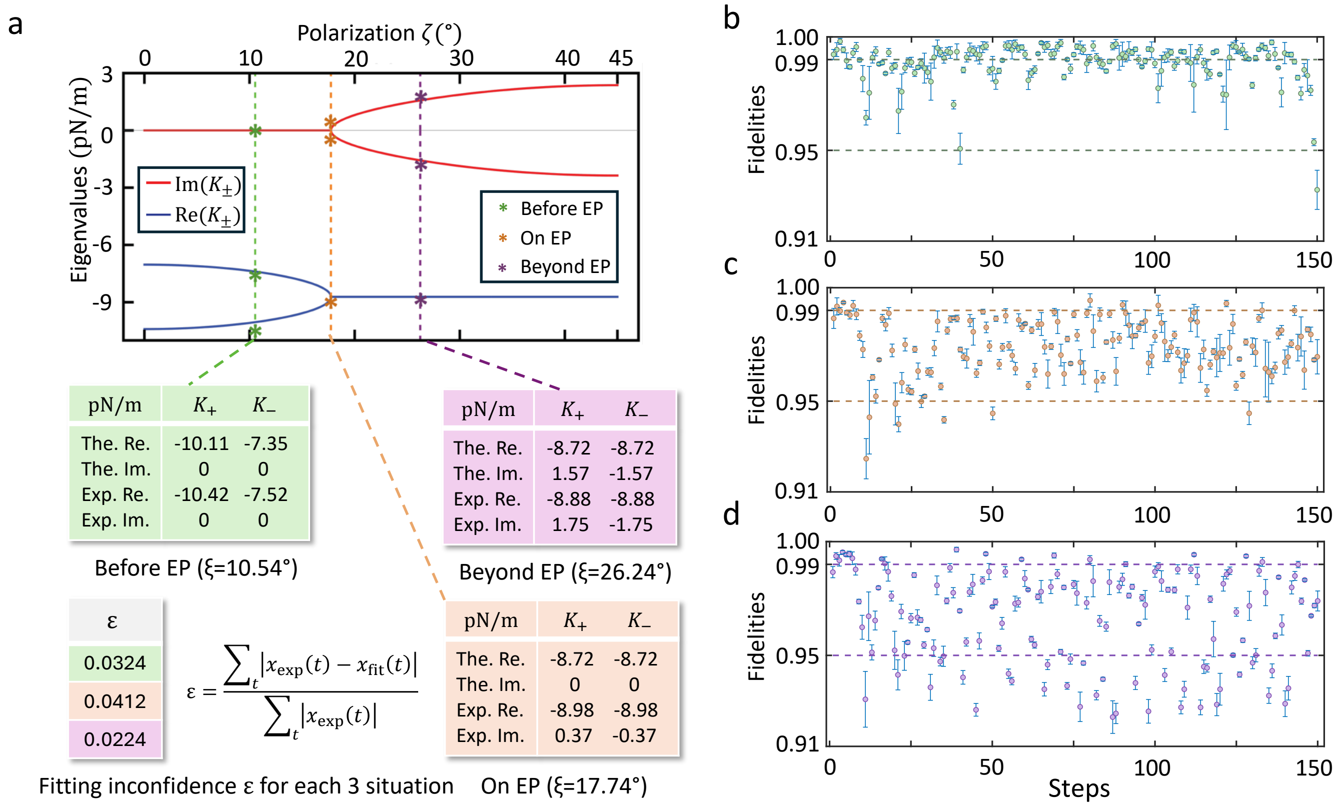

Figure S4. **The fitted eigenvalue $K_\pm$ and final state fidelity for each three situation.** **a**, The corresponding charts display the eigenvalues for both theoretical predictions (The.) and experimental fitting results (Exp.). The imaginary parts (Im.) and real parts (Re.) are shown by red and blue lines for theoretical predictions and colored stars for experimental fitting results. **b-d**, The fidelities of the measured final states for each 150 step of the experiment for each three situation.

Fig. S4b-d. In general, most infidelities here comes from the experimental error, including the coherent errors (mainly operation errors) and the incoherent errors (mainly dephasing errors). After receiving the density matrices from the experimental results, we proceed with the "Extraction of Experimental Results" section, which includes a step to diagonalize the density matrices. This process blocks the classical error transformation formula, therefore we have to perform randomly sampling to our multiple experimental results from the measurement of the final state, resulting in non-uniform error bars in Fig. 2 from the main text. As a reference, here we also present the data of our experimental results of velocities dynamics versus time as a reference in Fig. S5.

As the force matrix becomes more divergent, the fidelities of the final state decrease due to the increasingly rapid changes in the movement of the particle, but still maintain around 95%. The fidelities could be improved by setting shorter step lengths to reduce the influence of these rapid changes in more divergent situations.

We also present the exact fitted eigenvalues $K_\pm$ for each of the three situations in Fig. S4a. Additionally, as we fitted the experimental curve, we present the fitting inconfidence as $\epsilon = \frac{\sum_t |x_{\text{exp.}} - x_{\text{fit.}}|}{\sum_t |x_{\text{fit.}}|}$, and the three situations are 0.0324, 0.0412 and 0.0224 respectively, from which we can conclude that the fitted results are credible.

THREE-PARTICLE SIMULATION

Here we interpret the numerical simulations of the 1D three-particle optical-binding (OB) model. As it is a 1D model, the force matrix of the three particles is a 3×3 matrix $\tilde{\mathbf{K}}_3$. Considering the same expansion method we use in the previous single-particle experiment, we need additional three dimensions to simultaneously load the information of displacements and velocities. Therefore, the expanded force matrix $\tilde{\mathbf{K}}_3'$ is 6×6 that requires three system qubits to encode the information.

The numbers of ancillary qubits are determined by the number of elements in the LCU. In the three-particle OB model,

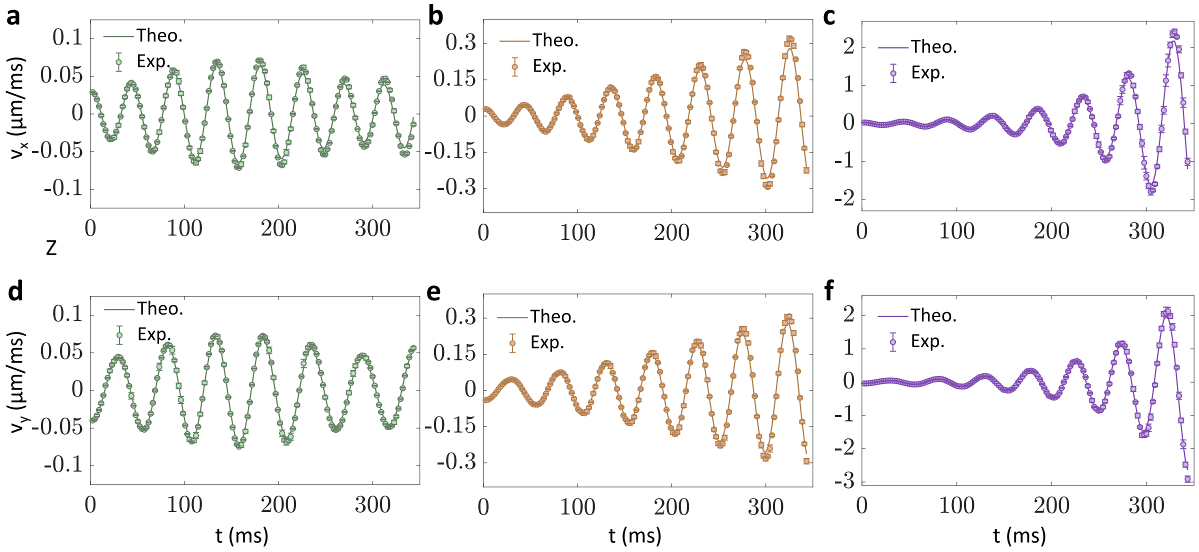

Figure S5. **Experimental results of velocities dynamics versus time.** For the three cases, stable, diverging, and unstable oscillations are observed, respectively in for velocities in x coordinate (**a-c**) and in y coordinate (**d-f**), consistent with predictions from non-Hermitian dynamics theory. The colored lines are theoretical predictions from numerical simulations and colored points are experimental results from quantum simulations. Error bars are deduced by performing randomly sampling to our multiple experimental results from the measurement of the final state.

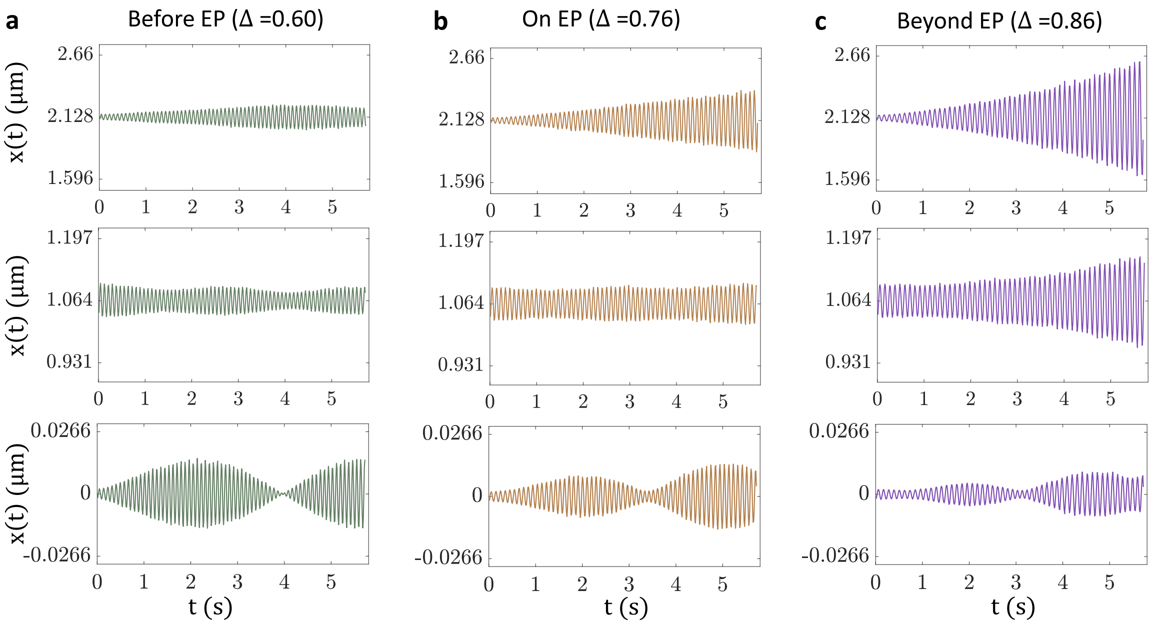

Figure S6. **Numerical simulations of the three-particle optical trapping model**. **a-c**, Displacements of the three particles over time before, on and beyond the EP. All three particles exhibit stable binding before the EP ($\Delta = 0.60$), linear divergence at the EP ($\Delta = 0.76$), and instability beyond the EP ($\Delta = 0.86$).

the force matrix can be written as

$$\tilde{\mathbf{K}}_3 = \begin{bmatrix} K_{11} & K_{12} & K_{13} \\ K_{21} & K_{22} & K_{23} \\ K_{31} & K_{32} & K_{33} \\ & & 1 \end{bmatrix}. \qquad (42)$$

The solution to the force equation $\ddot{\mathbf{X}} - \tilde{\mathbf{K}}_3\mathbf{X} = 0$ is $\mathbf{Z}(t) =$

$e^{\tilde{\mathbf{K}}'_3 t}\mathbf{Z}(0)$, where

$$\tilde{\mathbf{K}}'_3 = \begin{bmatrix} & & & & 1 & & & \\ & & & & & 1 & & \\ & & & & & & 1 & \\ & & & & & & & 1 \\ K_{11} & K_{12} & K_{13} & & & & & \\ K_{21} & K_{22} & K_{23} & & & & & \\ K_{31} & K_{32} & K_{33} & & & & & \\ & & & 1 & & & & \end{bmatrix} \tag{43}$$

is the expanded force matrix.

The next step is to perform LCU such that $\tilde{\mathbf{K}}'_3 = \sum_{i=1}^{n} c_i \tilde{\mathbf{U}}_i$. For the worst case, that is, all $K_{ij}|_{i,j=1,2,3}$ own no relationship with each other, we need to use two unitary matrices to encode one degree of freedom. In our case, knowing that no imaginary parts exist, at most $n = 13$ unitary matrices are needed. To illustrate this, we take the following matrix $\tilde{\mathbf{K}}'^1_3$ as an example:

$$\tilde{\mathbf{K}}'^1_3 = \begin{bmatrix} & & & & K_{s1}\tilde{\mathbf{I}}_4 \\ K_{11} & & & & \\ & K_{22} & & & \\ & & K_{33} & & \\ & & & K_{s1} & \end{bmatrix}, \tag{44}$$

where $K_{s1} = K_{11} + K_{22} + K_{33}$ and $\tilde{\mathbf{I}}_4$ is the 4×4 identity matrix. $\tilde{\mathbf{K}}'^1_3$ can be decomposed with 4 unitary matrices: where

$$\tilde{\mathbf{U}}_1 = \begin{bmatrix} & & & & \tilde{\mathbf{I}}_4 \\ 1 & & & & \\ & -1 & & & \\ & & -1 & & \\ & & & 1 & \end{bmatrix}, \tilde{\mathbf{U}}_2 = \begin{bmatrix} & & & & \tilde{\mathbf{I}}_4 \\ -1 & & & & \\ & 1 & & & \\ & & -1 & & \\ & & & 1 & \end{bmatrix}, \tilde{\mathbf{U}}_3 = \begin{bmatrix} & & & & \tilde{\mathbf{I}}_4 \\ -1 & & & & \\ & -1 & & & \\ & & 1 & & \\ & & & 1 & \end{bmatrix}, \tilde{\mathbf{U}}_4 = \begin{bmatrix} & & & & \tilde{\mathbf{I}}_4 \\ 1 & & & & \\ & 1 & & & \\ & & 1 & & \\ & & & 1 & \end{bmatrix}.$$

Similarly, we have:

$$\tilde{\mathbf{K}}'^2_3 = \frac{K_{12}}{2}\tilde{\mathbf{U}}_5 + \frac{K_{23}}{2}\tilde{\mathbf{U}}_6 + \frac{K_{31}}{2}\tilde{\mathbf{U}}_7 + \frac{K_{s2}}{2}\tilde{\mathbf{U}}_8,$$
$$\tilde{\mathbf{K}}'^3_3 = \frac{K_{13}}{2}\tilde{\mathbf{U}}_9 + \frac{K_{21}}{2}\tilde{\mathbf{U}}_{10} + \frac{K_{32}}{2}\tilde{\mathbf{U}}_{11} + \frac{K_{s3}}{2}\tilde{\mathbf{U}}_{12},$$

where

$$\tilde{\mathbf{U}}_5 = \begin{bmatrix} & & & & \tilde{\mathbf{I}}_4 \\ & 1 & & & \\ & & -1 & & \\ -1 & & & & \\ & & & 1 & \end{bmatrix}, \tilde{\mathbf{U}}_6 = \begin{bmatrix} & & & & \tilde{\mathbf{I}}_4 \\ & -1 & & & \\ & & 1 & & \\ -1 & & & & \\ & & & 1 & \end{bmatrix}, \tilde{\mathbf{U}}_7 = \begin{bmatrix} & & & & \tilde{\mathbf{I}}_4 \\ & -1 & & & \\ & & -1 & & \\ 1 & & & & \\ & & & 1 & \end{bmatrix}, \tilde{\mathbf{U}}_8 = \begin{bmatrix} & & & & \tilde{\mathbf{I}}_4 \\ & 1 & & & \\ & & 1 & & \\ 1 & & & & \\ & & & 1 & \end{bmatrix},$$

$$\tilde{\mathbf{U}}_9 = \begin{bmatrix} & & & & \tilde{\mathbf{I}}_4 \\ & & 1 & & \\ -1 & & & & \\ & -1 & & & \\ & & & 1 & \end{bmatrix}, \tilde{\mathbf{U}}_{10} = \begin{bmatrix} & & & & \tilde{\mathbf{I}}_4 \\ & & -1 & & \\ 1 & & & & \\ & -1 & & & \\ & & & 1 & \end{bmatrix}, \tilde{\mathbf{U}}_{11} = \begin{bmatrix} & & & & \tilde{\mathbf{I}}_4 \\ & & -1 & & \\ -1 & & & & \\ & 1 & & & \\ & & & 1 & \end{bmatrix}, \tilde{\mathbf{U}}_{12} = \begin{bmatrix} & & & & \tilde{\mathbf{I}}_4 \\ & & 1 & & \\ 1 & & & & \\ & 1 & & & \\ & & & 1 & \end{bmatrix},$$

and

$$\tilde{\mathbf{K}}_3'^2 = \begin{bmatrix} & & & & K_{s2}\tilde{\mathbf{I}}_4 \\ & K_{12} & & & \\ & & K_{23} & & \\ K_{31} & & & & \\ & & & K_{s2} & \end{bmatrix}, \tilde{\mathbf{K}}_3'^3 = \begin{bmatrix} & & & & K_{s3}\tilde{\mathbf{I}}_4 \\ & & K_{13} & & \\ K_{21} & & & & \\ & K_{32} & & & \\ & & & K_{s3} & \end{bmatrix}, \tag{45}$$

where $K_{s2} = K_{12}+K_{23}+K_{31}$ and $K_{s3} = K_{13}+K_{21}+K_{32}$. Now we have

$$\tilde{\mathbf{K}}_3' \to \tilde{\mathbf{K}}_3'^1 + \tilde{\mathbf{K}}_3'^2 + \tilde{\mathbf{K}}_3'^3 = \begin{bmatrix} & & & & K_s\tilde{\mathbf{I}}_4 \\ K_{11} & K_{12} & K_{13} & & \\ K_{21} & K_{22} & K_{23} & & \\ K_{31} & K_{32} & K_{33} & & \\ & & & K_s & \end{bmatrix}, \tag{46}$$

where $K_s = K_{s1} + K_{s2} + K_{s3}$. Noticing that the 4th term of both displacement and velocity are always zero, so the terms in 4th and 8th lines of the matrix does not cause any effect in the output vector. On the next step, one choice is to multiple the output displacements with $1/K_s$ like the same way we have done in one particle situation. And another choice is to introduce the 13th unitary matrix (since there is no difference for the needed qubit numbers we take between 12 matrices and 13 matrices):

$$\tilde{\mathbf{U}}_{13} = \begin{bmatrix} & & & & \tilde{\mathbf{I}}_4 \\ -1 & & & & \\ & -1 & & & \\ & & -1 & & \\ & & & 1 & \end{bmatrix}; \frac{\tilde{\mathbf{U}}_{13} + \tilde{\mathbf{U}}_4}{2} = \begin{bmatrix} & & & & \tilde{\mathbf{I}}_4 \\ 0 & & & & \\ & 0 & & & \\ & & 0 & & \\ & & & 1 & \end{bmatrix}.$$

It is easy to notice that an additional $(\tilde{\mathbf{U}}_{13}+\tilde{\mathbf{U}}_4)/2$ multiplied with $(1 - K_s)$ plus $\tilde{\mathbf{K}}_3'^1 + \tilde{\mathbf{K}}_3'^2 + \tilde{\mathbf{K}}_3'^3$ that we have already obtained forms precisely $\tilde{\mathbf{K}}_3'$. The final LCU of $\tilde{\mathbf{K}}_3'$ is thus

$$\begin{aligned} \tilde{\mathbf{K}}_3' =& \frac{K_{11}}{2}\tilde{\mathbf{U}}_1 + \frac{K_{22}}{2}\tilde{\mathbf{U}}_2 + \frac{K_{33}}{2}\tilde{\mathbf{U}}_3 + \frac{1 - K_s + K_{s1}}{2}\tilde{\mathbf{U}}_4 + \frac{K_{12}}{2}\tilde{\mathbf{U}}_5 + \frac{K_{23}}{2}\tilde{\mathbf{U}}_6 + \frac{K_{31}}{2}\tilde{\mathbf{U}}_7 + \frac{K_{s2}}{2}\tilde{\mathbf{U}}_8 \\ &+ \frac{K_{13}}{2}\tilde{\mathbf{U}}_9 + \frac{K_{21}}{2}\tilde{\mathbf{U}}_{10} + \frac{K_{32}}{2}\tilde{\mathbf{U}}_{11} + \frac{K_{s3}}{2}\tilde{\mathbf{U}}_{12} + \frac{1 - K_s}{2}\tilde{\mathbf{U}}_{13}, \end{aligned} \tag{47}$$

which needs four ancillary qubits.

The second-order of Taylor expansion is similar. Therefore,

four ancillary qubits are needed to complete the LCU for the three-particle OB case. In the main text, we only show the envelop of the simulation results. Here, we present the exact simulation results including the rapid oscillations in Fig. S6.

* These authors contributed equally to this work.
† wuzh3@sustech.edu.cn
‡ ludw@sustech.edu.cn

[1] Xu, Y.-L. Electromagnetic scattering by an aggregate of spheres. *Applied optics* **34**, 4573–4588 (1995).
[2] Stein, S. Addition theorems for spherical wave functions. *Quarterly of Applied Mathematics* **19**, 15–24 (1961).
[3] Cruzan, O. R. Translational addition theorems for spherical vector wave functions. *Quarterly of Applied Mathematics* **20**, 33–40 (1962).
[4] Xu, Y.-L. Efficient evaluation of vector translation coefficients in multiparticle light-scattering theories. *Journal of Computational Physics* **139**, 137–165 (1998).
[5] Mishchenko, M. I., Hovenier, J. W. & Travis, L. D. Light scattering by nonspherical particles: theory, measurements, and applications. *Measurement Science and Technology* **11**, 1827–1827 (2000).
